# Effects of Syringaldehyde/Gum Arabic Composite Chitosan Thermochromic Microcapsules on the Coating Properties of Basswood Surface

**DOI:** 10.3390/polym18162017

**Published:** 2026-08-20

**Authors:** Wenjing Chang, Jingyi Hang, Xiaoxing Yan

**Affiliations:** 1 Co-Innovation Center of Efficient Processing and Utilization of Forest Resources, Nanjing Forestry University, Nanjing 210037, China; 1127096308@njfu.edu.cn (W.C.);; 2 College of Furnishings and Industrial Design, Nanjing Forestry University, Nanjing 210037, China

**Keywords:** thermochromic microcapsules, chitosan, crystal violet lactone, UV coating

## Abstract

Thermochromic wood coatings hold promising application prospects, yet conventional thermochromic microcapsules are limited by monotonous color transitions and non-environmentally friendly wall materials. In this study, two formaldehyde-free thermochromic microcapsules were prepared via spray drying using crystal violet lactone (CVL) and bisphenol A (BPA) as the core system, with chitosan–syringaldehyde (SA-MCs, decyl alcohol as solvent, Schiff base crosslinking) and chitosan–gum Arabic (GA-MCs, lauryl alcohol as solvent, electrostatic complex coacervation) as the wall materials, respectively. These microcapsules were incorporated into basswood ultraviolet (UV) coatings at mass fractions of 1%, 3%, 5%, 7%, and 9%. With increasing microcapsule content, the gloss of both coatings decreased progressively, roughness increased gradually, and the color-changing amplitude, expressed as the color difference (Δ*E*), was continuously enhanced. At 9% addition, the SA-MC coating exhibited a moderate transition from light yellow to yellow-green (Δ*E* = 7.2), while the GA-MC coating displayed a dramatic reversible change from deep blue to light gray (Δ*E* = 42.4), both with good reversibility. In terms of mechanical properties, SA-MCs exhibited higher hardness, both systems achieved an impact resistance grade of 3, and GA-MCs demonstrated superior adhesion. After 24 h of short-term UV accelerated aging, GA-MCs still maintained a higher thermochromic response, albeit with more severe gloss loss. In summary, GA-MCs are superior in color-changing amplitude and adhesion, while SA-MCs offer advantages in gloss retention and hardness, providing a reference for material selection in the application of smart wood finishing.

## 1. Introduction

Thermochromic materials are defined as a class of functional materials that are capable of undergoing color transitions in response to changes in environmental temperature [1,2]. Based on their composition and physicochemical properties, thermochromic materials are classified into three categories: inorganic thermochromic materials, organic thermochromic materials, and liquid crystal thermochromic materials. At present, organic reversible thermochromic materials based on the electron transfer mechanism have been widely applied [3,4,5]. These materials undergo molecular configuration changes and electron transfer at specific temperatures, by which color transitions occur. They are generally composed of three components: a leuco dye, a color developer, and a solvent [6]. Among these, the leuco dye is responsible for determining the basic color, the color developer is used to regulate the color depth, and the solvent influences the color-changing temperature. Overall, such materials are characterized by the advantages of low color-changing temperature, good reversibility, and sensitive response [7].

Crystal violet lactone (CVL), as a typical triarylmethane leuco dye, is widely used in thermochromic systems due to its sensitive color change, vivid color, and excellent reversibility [8,9,10]. Its color-changing mechanism involves the reversible ring-opening and ring-closing of the lactone ring [11,12]. At low temperature, the ring is closed, conjugation is weak, and the material appears light in color. Upon heating, the ring opens, forming a conjugated system that develops color. Upon cooling, the ring closes, and the color fades. By adjusting the types and proportions of the color developer and solvent, the color-changing temperature and color-changing amplitude of the CVL system can be effectively regulated, making it an ideal leuco dye for constructing low-temperature reversible thermochromic systems [13,14,15,16]. Wang et al. modified the CVL-based thermochromic material with methyl red, and a color change from blue-purple to orange-red was successfully achieved [17]. Li et al. realized a reversible cyclic thermochromic transition of TLW from red to purplish-red at 38–52 °C and back to red upon cooling [18].

However, thermochromic materials are generally characterized by poor acid and alkali resistance and susceptible to external environmental interference, which limits their direct application. Microencapsulation technology, by encapsulating functional substances within wall materials, can effectively protect the core materials from external factors, improve material stability, and broaden the scope of application [19,20]. Zou et al. synthesized thermochromic microcapsules with CVL as the core material and urea-formaldehyde resin as the wall material [21]. After the optimal microcapsules were added to a UV-curable system at a proportion of 10%, the UV-curable coating was successfully endowed with reversible thermochromic functionality, while good mechanical and optical properties were also maintained, which broadened the application prospects of microcapsules in UV-curable materials. Wang et al. used CVL as the color former, bisphenol A (BPA) as the developer, and methyl red-modified octanediol as the solvent, and reversible thermochromic microcapsules with excellent stability were prepared by in situ polymerization [22]. These microcapsules enabled cotton and polyester fabrics to undergo a reversible transition from blue-purple to orange-red at 30–40 °C, while good washability was also imparted to the fabrics, which provided a feasible approach for enriching the color system of thermochromic textiles.

However, the above studies mostly employed synthetic wall materials such as urea-formaldehyde resin, causing environmental safety issues such as formaldehyde emission [23,24]. Moreover, the color changes were mostly limited to a single color system, making it difficult to satisfy the demand for rich color variations in wood products. In response to the environmental issues associated with synthetic wall materials, natural polymer materials have attracted considerable attention due to their good biocompatibility, biodegradability, and environmental friendliness. Chitosan (CS) is a widely available renewable natural polymer. Abundant amino and hydroxyl groups are present on its molecular chains, which enable the formation of stable wall structures with various crosslinking agents [25,26]. Syringaldehyde (SA) crosslinks with CS via Schiff base reaction, while gum Arabic (GA) complexes with CS through electrostatic coacervation of anions and cations. As natural raw materials, SA is a natural monoaldehyde and GA is a natural anionic polysaccharide [27,28,29,30]. The composite wall materials formed from them are colorless and transparent, and will not interfere with the thermochromic performance of the CVL system. Therefore, the use of CS-based natural wall materials as a substitute for synthetic resins is considered an effective approach to enhancing the environmental friendliness of microcapsules.

UV-curable coatings have become one of the mainstream technologies for wood surface finishing, owing to their advantages of fast curing speed, high film-forming quality, and low volatile organic compound (VOC) emissions [31,32]. The application of the above thermochromic microcapsules to UV coatings on wood surfaces can endow wood products with intelligent reversible color-changing functionality while retaining the advantages of the rapid curing process. Basswood, due to its clear grain, uniform texture, light color, and excellent processing properties, is commonly used as a substrate in interior decoration and solid wood furniture. Its light-colored surface provides a clear visual display basis for the color changes of functional coatings [33,34,35]. However, existing thermochromic microcapsules are mostly limited to lightness changes within a single color system. Therefore, expanding the color-changing spectrum of thermochromic microcapsules and achieving multi-tone reversible transformation are of great significance for enhancing the intelligent decorative effects of wood products.

In this study, CVL was used as the leuco dye and BPA as the color developer. Two thermochromic compounding systems were constructed with decyl alcohol (DA) and lauryl alcohol (LA) as solvents, respectively. Thermochromic microcapsules were fabricated via two distinct green encapsulation strategies using formaldehyde-free wall materials: Schiff base crosslinking with SA and electrostatic complex coacervation with GA (SA-MCs and GA-MCs). The influences of the two crosslinking formation mechanisms on the particle size properties of microcapsules were compared. Furthermore, the two types of microcapsules were separately incorporated into basswood UV coatings at varying mass fractions. The variation rules of thermochromic effect, optical performance, surface roughness, mechanical properties and short-term UV stability of coatings under single addition mode were systematically compared, and the respective advantages and disadvantages of the two microcapsules were clarified. This work provides experimental support for the formulation selection of thermochromic wood coatings.

## 2. Experiment

### 2.1. Experimental Materials

The experimental materials and equipment are listed in Tables S1 and S2. The basswood panels used in the experiment measured 50 mm × 50 mm × 5 mm and were purchased from Beijing Yidimei Model Co., Ltd. (Beijing, China). The UV topcoat used in the experiment was a scratch-resistant topcoat (model 258XXX511UV) manufactured by Jiangsu Haitian Technology Co., Ltd. (Jurong, China). Its main components included polyurethane acrylate resin, propylene glycol diacrylate, hexanediol diacrylate, photoinitiator, matting agent, defoamer, leveling agent, etc., with a solid content greater than 98.0%.

### 2.2. Microcapsule Preparation Method

Based on literature review and preliminary experimental results, the optimal preparation methods for SA-MCs and GA-MCs were determined in this study [36,37,38]. For the SA-MC system, CS and SA were used as the wall materials, with CVL and BPA as the core material system and DA as the solvent. For the GA-MC system, CS and GA were used as the wall materials, with CVL and BPA as the core material system and LA as the solvent. The detailed preparation procedures were as follows.

(1) SA-MC Preparation Method

CS (1.000 g) was dissolved in 99.000 mL of 1% acetic acid at 50 °C with stirring at 1000 r/min to obtain the wall material solution. The core material was prepared with a CVL: BPA: DA mass ratio of 1:3:50. DA (5.000 g) was melted at 30 °C, followed by the addition of BPA (0.300 g) and CVL (0.100 g). The mixture was stirred at 400 r/min and 50 °C for 1.5 h to form the core material emulsion, which was then cooled to room temperature. For emulsification, Tween 80 (0.148 g) and Span 80 (0.132 g) were dissolved in 19.72 mL of distilled water, and 1.40 g of the core emulsion was added. The mixture was stirred at 60 °C for 20 min at 1000 r/min, followed by ultrasonic treatment for 5 min. The wall material solution was then added dropwise to the core emulsion at 35 °C with stirring at 600 r/min, and the pH was adjusted to 5 with acetic acid. After mixing for 1 h, SA (0.400 g) dissolved in 10.000 mL of anhydrous ethanol was added as the crosslinker, and the reaction proceeded at 60 °C for 2 h. After standing at room temperature for 1 h, the solution was spray-dried at an inlet temperature of 120 °C and a feed rate of 120 mL/h to obtain SA-MC powder.

**Figure 1:**
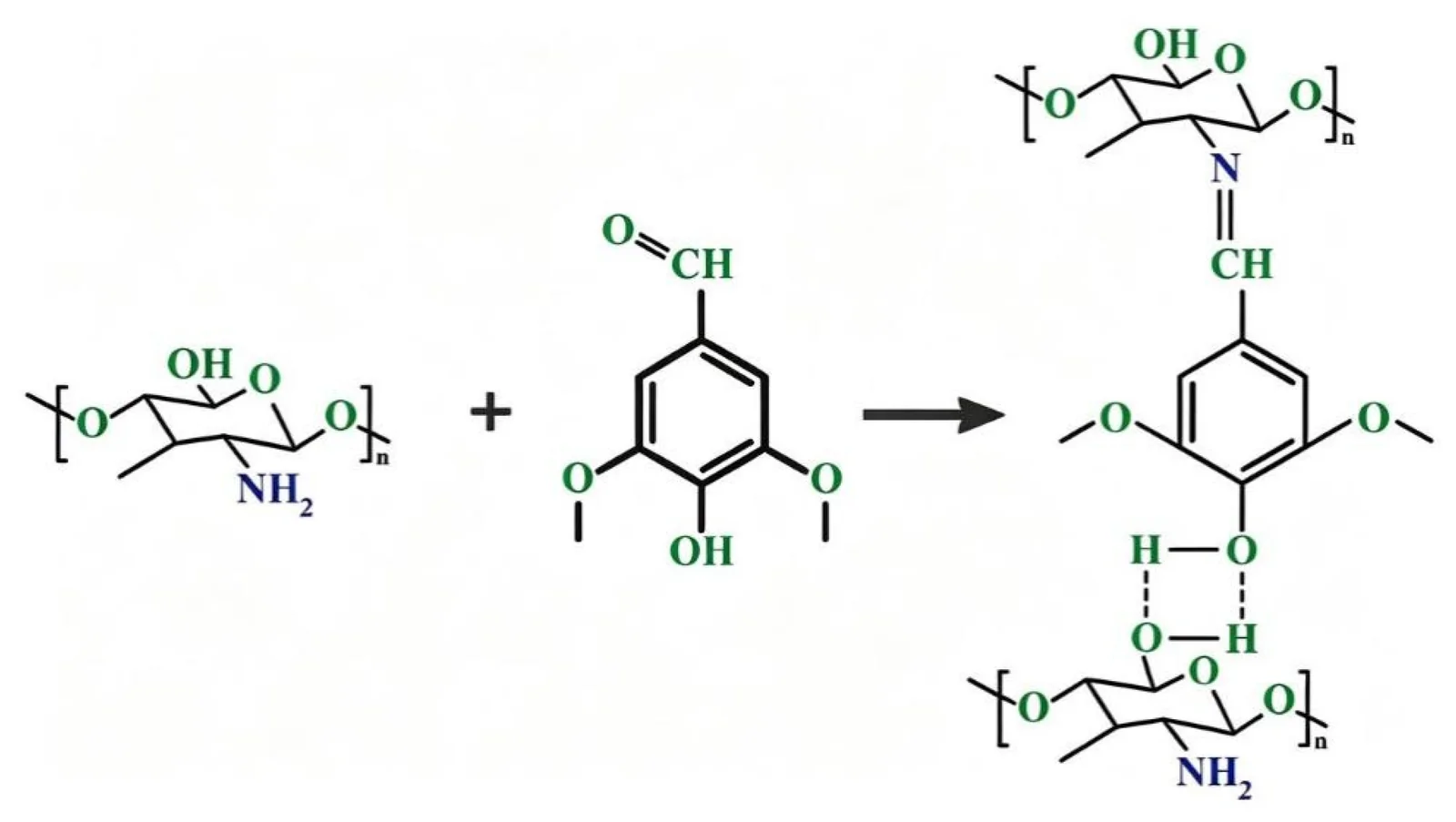
Crosslinking mechanism of CS with SA.

Figure 1 illustrates the crosslinking mode between CS and SA. The structural formula of SA contains both aldehyde and hydroxyl groups. The carbonyl carbon in SA carries a positive charge and readily reacts with nucleophilic reagents such as CS. A Schiff base reaction readily occurs between the aldehyde group of SA and the amino group of CS, forming imine bonds. Meanwhile, hydrogen bonds can form between the hydroxyl groups of SA and the hydroxyl groups of CS. In this manner, chemical crosslinking (Schiff base) is formed on one side, while physical crosslinking (hydrogen bonding) is formed on the other, achieving relatively strong chemical-physical crosslinking.

(2) GA-MC Preparation Method

CS (0.300 g) was dissolved in 29.700 mL of 1% acetic acid at 50 °C with stirring at 1000 r/min to obtain the wall material solution. The core material was prepared with a CVL:BPA:LA mass ratio of 1:2:60. LA (12.000 g) was melted at 30 °C, followed by the addition of BPA (0.400 g) and CVL (0.200 g). The mixture was stirred at 600 r/min and 60 °C for 1.5 h to form the core emulsion, which was then cooled to room temperature. For emulsification, GA powder (1.200 g) was dissolved in 38.800 g of deionized water at 50 °C for 1 h to obtain a 3% GA solution, into which 1.500 g of the core material was added and stirred at 1600 r/min for 1 h, followed by ultrasonic treatment for 5 min. The wall material solution was then added dropwise to the core emulsion at 50 °C with stirring at 800 r/min, and the pH was adjusted to 4 with acetic acid. After mixing for 30 min, the solution was diluted 1:1 with deionized water and stirred at 25 °C for another 30 min. After standing at room temperature for 1 h, the solution was spray-dried at an inlet temperature of 105 °C and a feed rate of 120 mL/h to obtain GA-MC powder.

It should be clarified that the term “formaldehyde-free” only describes the shell raw materials of the as-prepared microcapsules, rather than the entire UV coating system.

### 2.3. Coating Application Method

The basswood panels were coated manually with two primer coats and two topcoats. The substrates were pretreated by sanding with 800-grit sandpaper to remove surface burrs and dust, ensuring good substrate condition for coating adhesion. Two microcapsule types were incorporated into the UV primer at mass fractions of 0, 1%, 3%, 5%, 7%, and 9%. Each coating layer was precisely controlled at 0.500 g total mass, with a single-layer application amount of 160 g/m^2^ to account for brush adhesion and application loss. The 0.500 g UV primer was uniformly applied onto the substrate. After a 10 min leveling period, the panels were cured in a single-lamp UV unit at a conveyor speed of 0.1 m/s for 1 min. The primer application and curing were repeated, followed by two topcoats applied and cured under the same conditions. The addition amounts of microcapsules and coatings are listed in Table 1.

### 2.4. Test and Characterization

(1) Coating microstructure

The microstructure of microcapsule powders and the surface and cross-sectional morphologies of coating films were characterized by optical microscopy (OM) and scanning electron microscopy (SEM).

Particle size analysis: SEM images of microcapsule powders were captured in multiple random fields of view. ImageJ software (1.51j8) was used to measure and calculate the particle size distribution histogram and average particle size.

Coating thickness measurement: Cross-sectional SEM images of coated basswood panels were employed for testing. Five evenly distributed positions on each cross-section were selected to measure the total coating thickness (distance from the coating–wood interface to the outermost coating surface) via ImageJ software, and the average value was adopted for each sample.

(2) Chemical composition characterization of coatings

The chemical compositions of the microcapsules and coating films were tested and characterized using Fourier transform infrared spectroscopy (FTIR). For the FTIR test, the microcapsule powder was pressed into thin pellets using a powder pressing machine.

(3) Optical performance testing of coatings

Color difference (Δ*E*): The samples were placed in a refrigerator or on a constant-temperature heating plate to reach the desired temperature. A handheld laser-sensing thermometer was then used to measure the surface temperature of the samples. During the measurement, the coating surface temperature was first measured using the laser-sensing thermometer. After the set temperature was reached, the test was performed using a portable color difference meter according to the test method specified in GB/T 11186.3-1989 [39]. After the color difference meter was calibrated, the test aperture was aligned with the coating surface to be tested, and the test button was pressed. The *L*, *a*, and *b* values were recorded. The *L* value represents the lightness of the color, with higher values indicating higher brightness. The *a* value represents the chromaticity in the red-green direction, with positive values indicating a tendency toward red and negative values indicating a tendency toward green. The *b* value represents the chromaticity in the yellow-blue direction, with positive values indicating a tendency toward yellow and negative values indicating a tendency toward blue. The *L*, *a*, and *b* values represent the values of the test sample after temperature change, while *L*_0_, *a*_0_, and *b*_0_ represent the values of the sample at the initial test temperature (−10 °C). Each set of data was measured four times, and the average value was taken. The formula for calculating the color difference Δ*E* between two points is shown in Formula (1) below.

∆*E =* [(*L* − *L*_0_)^2^ + (*a* − *a*_0_)^2^ + (*b* − *b*_0_)^2^]^1/2^ (1)

Gloss: The test was conducted according to GB/T 4893.6-2013 [40]. A portable gloss meter was used for the test. After the instrument was calibrated, the test aperture was aligned with the coating to be tested, and the gloss values of the coating at incident angles of 20°, 60°, and 85° were recorded from the screen. *G*_1_ represents the surface gloss of the coating after microcapsule addition, and *G*_0_ represents the surface gloss of the coating film without microcapsules. The gloss loss rate *G* was calculated according to Formula (2).

(2) $$G=\frac{G_{0}-G_{1}}{G_{0}}\;\times \;100\%$$

Reflectance: The reflectance of the coating film reflects its ability to reflect incident light, that is, the percentage of the incident luminous flux that is reflected by the coating film surface. The reflectance of the coating film in the visible light range of 350–780 nm was characterized using a UV-visible spectrophotometer, in order to evaluate the optical reflectance properties of the coating film.

(4) Mechanical performance testing of coatings

Roughness: The roughness of the samples was measured using a fine roughness tester. Each sample was subjected to three replicate tests. The sampling length was set to 0.800 mm, and the evaluation length was 4.000 mm, covering five segments of sampling length. During testing, the specimen was fixed on the sample platform, and the probe position was adjusted via the knob to make contact with the coating surface. The stylus scanned the surface to acquire the raw contour signals, followed by automatic calculation of all roughness parameters.

Hardness: The test was conducted according to GB/T 6739-2006 [41]. A pencil hardness tester was used to test the hardness of the coating films. Pencils with hardness grades ranging from 6H to 6B were selected, and the pencil leads were sharpened to flat end faces of approximately 4 mm. During the test, the pencil was fixed in the hardness tester with a specified load of 750 g so that the tip remained parallel to the coating film surface, and was pushed forward at a speed of approximately 0.5 mm/s for a distance of 1 cm. The test was conducted in order from soft to hard, and the damage on the coating film surface was observed. When no damage was observed on the coating film surface, the hardness of the pencil was taken as the maximum hardness of the coating film. Each sample was tested three times at different positions, and the reported hardness grade represents the mode (the most frequently occurring grade) of the three replicates.

Impact resistance: The test was conducted according to GB/T 4893.9-1992 [42]. A coating impact tester was used to test the impact performance of the samples. The sample was horizontally fixed on the base, and an impact block with a mass of 1.0 kg was dropped from a set height to impact the coating surface. The presence of cracks or peeling was observed. Before the experiment, the coating without microcapsules was tested first. The impact heights were set at 10 mm, 25 mm, 50 mm, 100 mm, 200 mm, and 400 mm, and each height was tested five times from low to high until Grade 5 cracks were produced on the impacted sample. It was found that the coating without microcapsules produced Grade 5 cracks at a height of 50 mm. Therefore, the height was set to 50 mm, and the impact resistance of each coating was tested at this height. Each coating was impacted five times, and the results were compared with Table S3. The original distribution of the five impact grades was recorded, and the mode was reported as the final impact resistance grade.

Adhesion: The test was conducted according to GB/T 4893.4-2013 [43]. A cross-cut tester was used to make right-angle grids on the coating surface in both longitudinal and transverse directions. Adhesive tape was then applied over the grids and peeled off. The detachment of the coating on the surface was observed after tape removal, and the adhesion was graded according to the detached area. The adhesion grades ranged from 0 to 5, corresponding to detached areas of 0%, 5%, 15%, 35%, 55%, and above 60%, respectively.

(5) Short-term UV aging test

The short-term UV aging test of the coatings was conducted according to GB/T 1865-2009 in a UV yellowing resistance test chamber [44]. The xenon lamp irradiance was set to 0.55 W/m^2^, and the black panel temperature was set to 60 °C. The coatings incorporated with thermochromic microcapsules were placed in the UV yellowing resistance test chamber and removed after 24 h of continuous irradiation. This exposure period was selected as an accelerated screening test to evaluate the early-stage aging response of the coatings, providing a comparative basis for distinguishing the degradation behavior between the two microcapsule systems. The chromatic values and gloss of the coating surfaces were then measured to analyze the initial effects of photoaging on the color-changing performance and optical properties.

## 3. Results and Discussion

### 3.1. Microstructure and Chemical Composition Analysis

(1) Microstructure characterization

**Figure 2:**
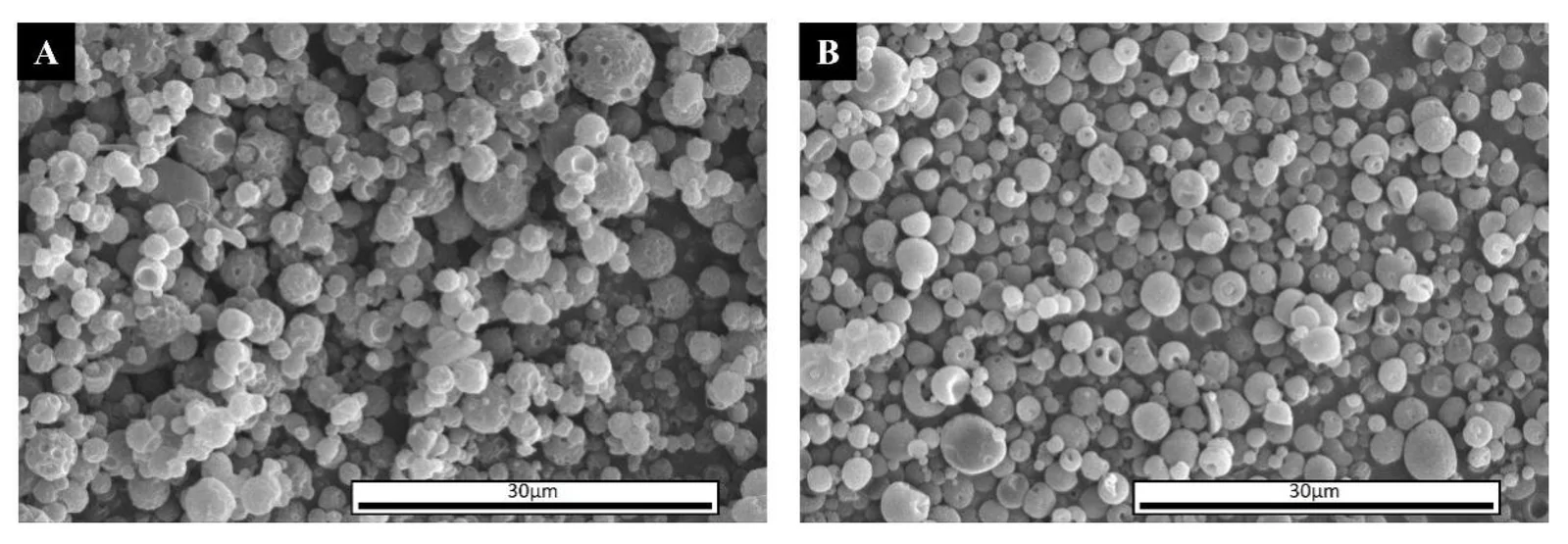
SEM images of two types of thermochromic microcapsule powders: (**A**) SA-MCs, (**B**) GA-MCs.

**Figure 3:**
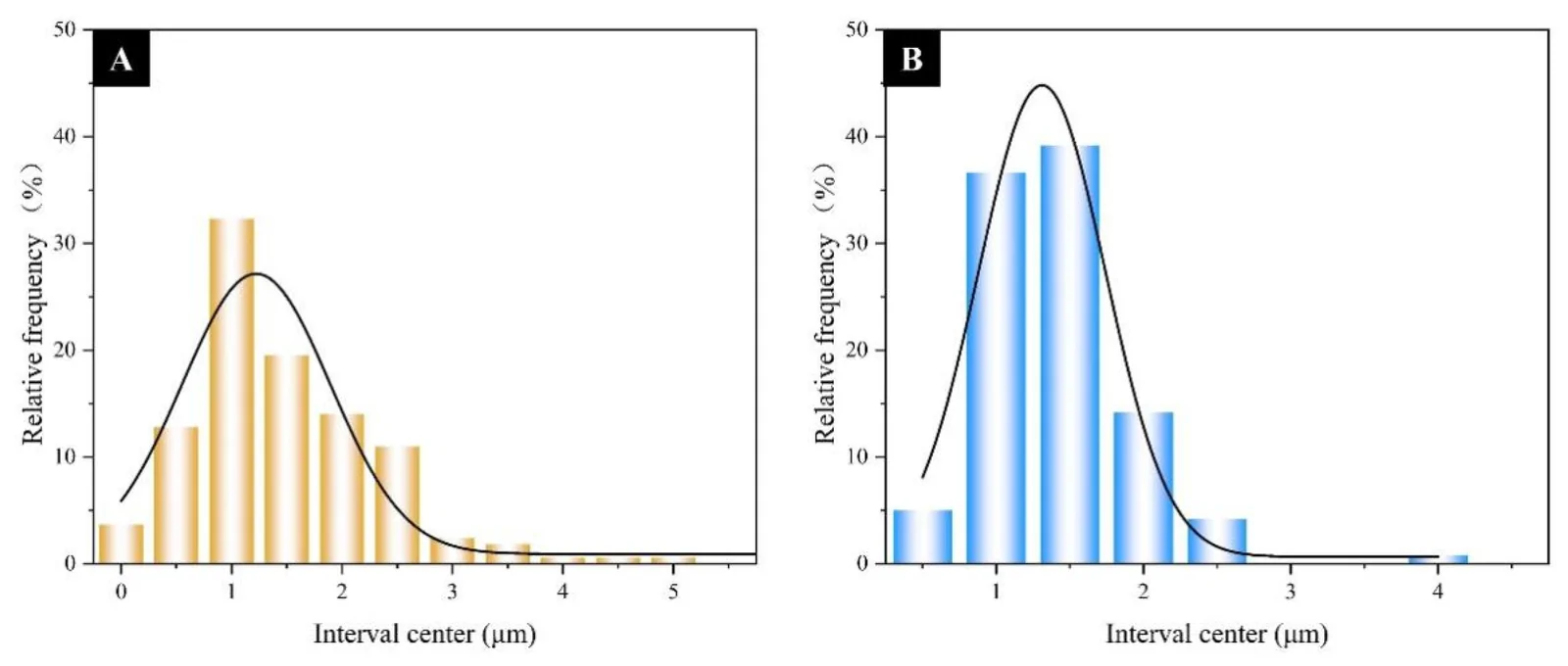
Particle size distributions of two types of thermochromic microcapsule powders: (**A**) SA-MCs, (**B**) GA-MCs.

Figure 2 shows the SEM morphologies of the two thermochromic microcapsule powders. SA-MCs exhibited a relatively regular spherical shape with slight surface depressions caused by spray drying, whereas GA-MCs displayed good sphericity with a smoother and denser surface. Figure 3 presents the particle size distributions statistically analyzed from the SEM images. The average particle size of SA-MCs was 1.745 μm, with a relatively broad distribution range (0.5 to >5.0 μm). The relative frequency showed a main peak at 1.0 μm, but maintained a high proportion in the 2.0–3.0 μm range, with a pronounced right tail, indicating the presence of a certain number of larger particles and soft agglomerates in the system. GA-MCs had an average particle size of 1.655 μm, close to that of SA-MCs, but exhibited a significantly narrower distribution (0.5–3.0 μm). The main peak was located near 1.5 μm, and the proportion of particles >3.0 μm was extremely low, demonstrating better monodispersity.

Although the difference in average particle size between the two was less than 5%, the significant difference in particle size distribution directly affected their dispersion behavior in the coating. The broad distribution of SA-MCs meant that a small number of large particles (>3 μm) were more prone to form local agglomeration in the coating and became stress concentration points during the curing process, whereas the narrow distribution and uniform particle size of GA-MCs were conducive to forming a more uniform and dense packing structure in the coating.

**Figure 4:**
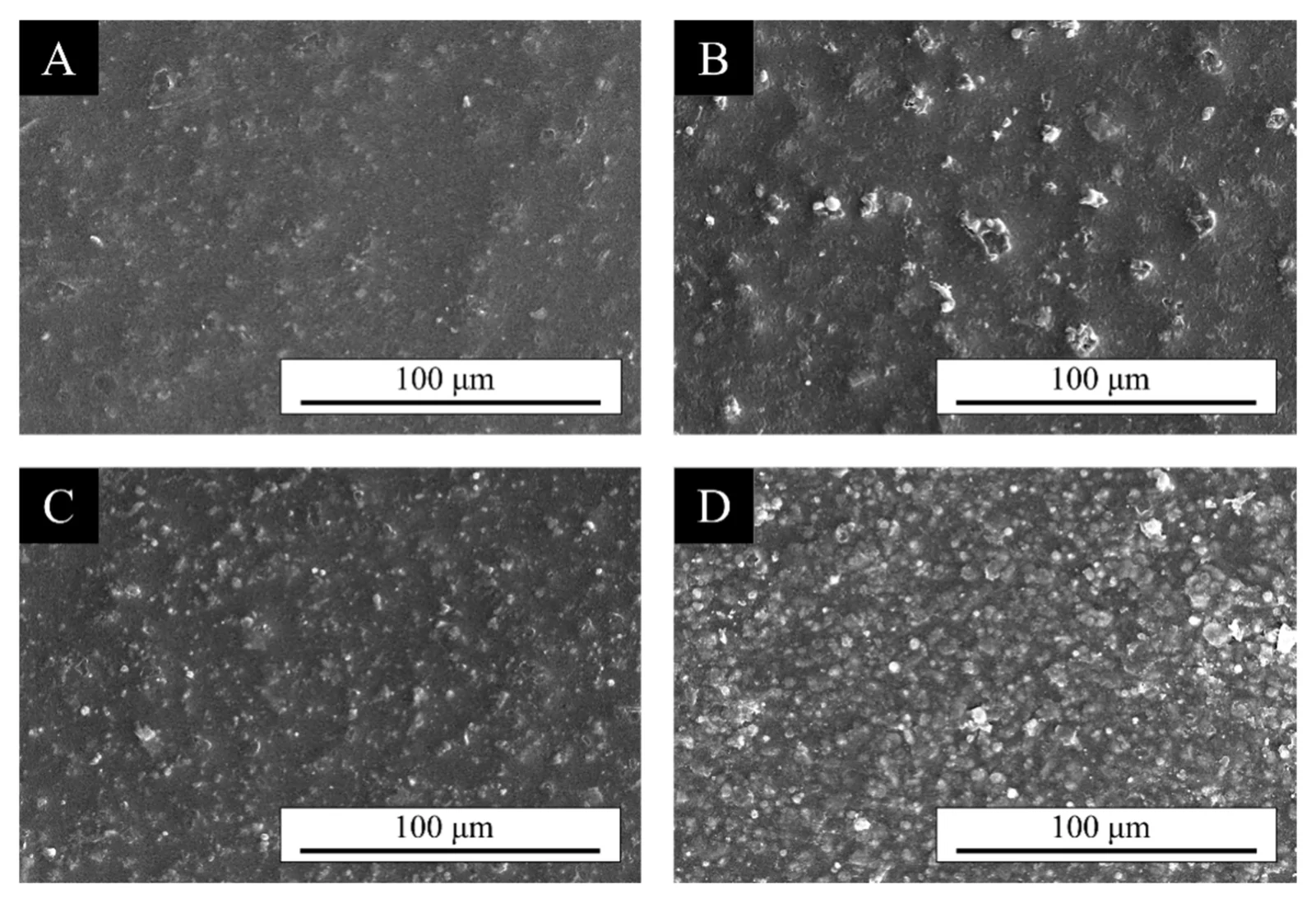
SEM images of surface coatings on basswood with different contents of SA-MCs: (**A**) 0%, (**B**) 1%, (**C**) 5%, (**D**) 9%.

**Figure 5:**
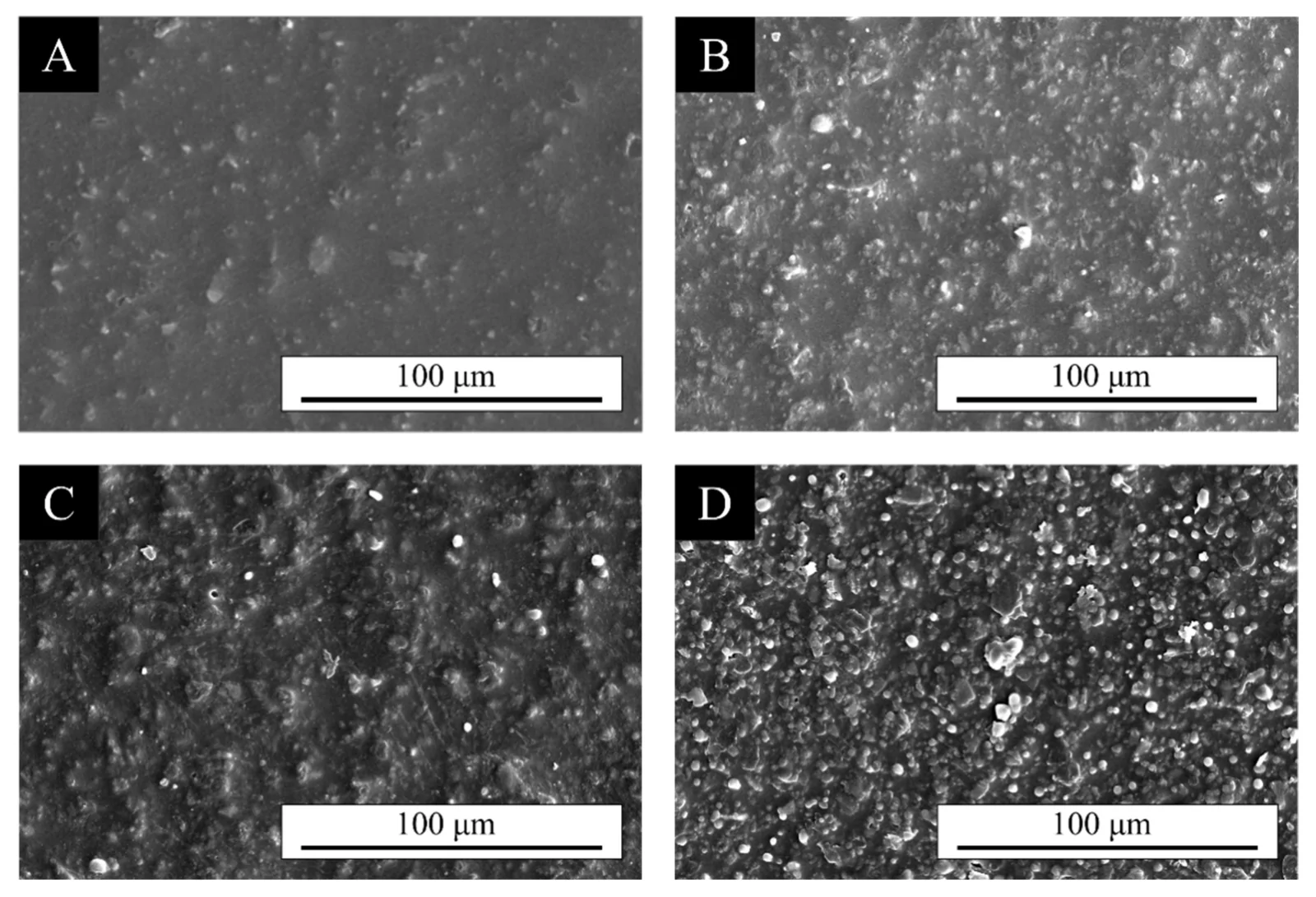
SEM images of surface coatings on basswood with different contents of GA-MCs: (**A**) 0%, (**B**) 1%, (**C**) 5%, (**D**) 9%.

Figure 4 and Figure 5 show the SEM images of basswood surface coatings with different contents of SA-MCs and GA-MCs, respectively. As the microcapsule content increased gradually from 0% to 9%, the surfaces of both coating types transformed from originally smooth and flat to structures with obvious particle distribution and progressively increased surface roughness. In the 1–5% content range, SA-MCs were mainly distributed on the coating surface as discrete particles; however, due to their broad particle size distribution, agglomeration caused by large particles could be observed in local areas (Figure 4B,C). When the content increased to 9%, the number of particles increased significantly, surface irregularities became pronounced, the packing between particles was loose, and numerous gaps existed (Figure 4D). In contrast, GA-MCs, owing to their narrow particle size distribution and uniform particle size, exhibited a more uniform dispersion state at all contents; at 9% content, the particles formed a relatively dense packing layer on the coating surface, with smaller interparticle gaps and a tighter surface microstructure (Figure 5D). This difference was consistent with the gloss measurements: the gloss of GA-MC coatings decreased more significantly (the 60° gloss loss rate reached 80.58% at 9% content), which could be attributed to the denser surface particle coverage enhancing the diffuse reflection and scattering of light.

**Figure 6:**
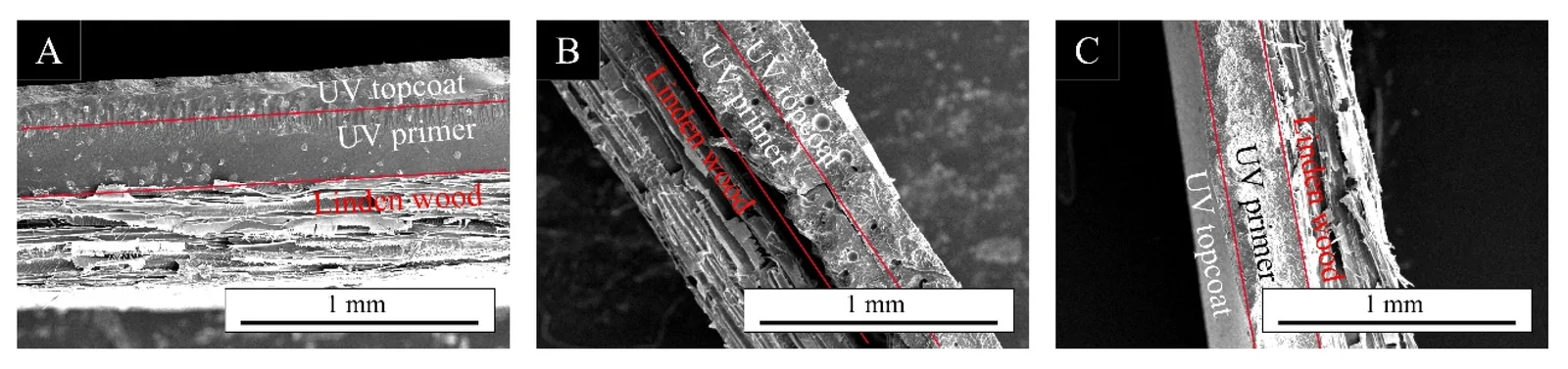
Longitudinal section of basswood boards: (**A**) #0, (**B**) #5, (**C**) #10.

Figure 6 shows the SEM microstructures of the longitudinal cross-section of basswood panels. For the 0# sample without microcapsules, the total coating thickness was approximately 0.49 mm. The cross-sections of the UV topcoat and primer layers were flat and dense; no obvious bubbles, cracks, or voids were observed inside the paint film, and the interface between the coating and the basswood substrate was tightly bonded. For the 5# sample with 9% SA-MCs, the coating thickness was approximately 0.40 mm. Numerous dispersed spherical bubbles and microcapsule particles were observed in the topcoat layer, and obvious cracks and interfacial delamination appeared in some areas; the overall flatness and densification of the paint film were significantly reduced. For the 10# sample with 9% GA-MCs, the coating thickness was approximately 0.27 mm. The microcapsules were relatively uniformly distributed in both the topcoat and primer layers; almost no obvious bubbles were observed inside the coating, and no cracking or delamination occurred.

(2) Chemical composition testing

**Figure 7:**
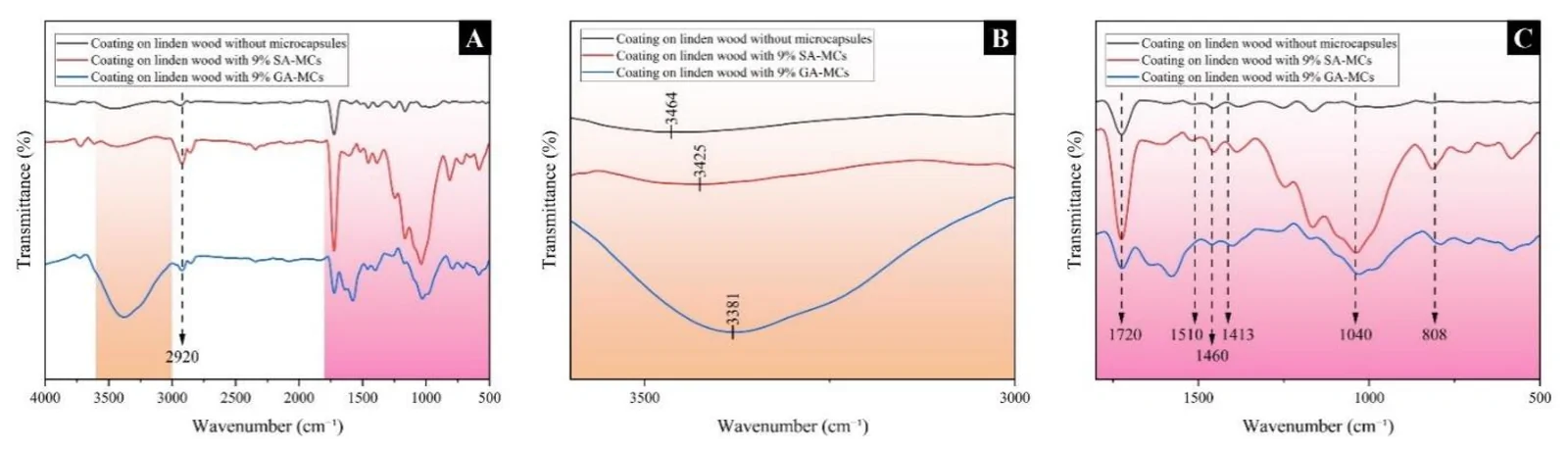
FTIR spectra of the UV-coated basswood boards: (**A**) full spectra, (**B**) enlarged region of 3600–3000 cm^−1^, (**C**) enlarged region of 1800–500 cm^−1^.

Figure 7 presents the FTIR spectra of UV coatings on basswood surfaces. Owing to the mass fraction of microcapsules in the coating being only 9%, and with the wall material being natural polysaccharides and the core material a low-concentration organic dye system, their characteristic absorption signals were relatively weak. Some peaks were masked by the strong absorption of the UV polyurethane acrylate resin matrix; therefore, the coating spectra overall retained the main framework of the UV coating system.

In the 3600–3000 cm^−1^ region (Figure 7B), the blank coating without microcapsules exhibited a broad absorption band at 3464 cm^−1^, which was mainly attributed to the O–H stretching vibrations of the polyurethane/acrylate components in the UV resin and a minor contribution from N–H stretching vibrations. After the addition of 9% SA-MCs, this peak shifted to 3425 cm^−1^; after the addition of 9% GA-MCs, it shifted further to 3381 cm^−1^, with the peak shape broadening significantly. The systematic redshift in the 3600–3000 cm^−1^ region (blank coating: 3464 cm^−1^ → SA-MC coating: 3425 cm^−1^ → GA-MC coating 3381 cm^−1^) warrants attention, as this spectral change may arise from multiple contributing factors. On one hand, the microcapsule wall materials (CS, GA, SA) are natural polymers rich in polar groups such as –OH, –NH_2_, and –COO^−^, which have the potential to form intermolecular hydrogen bonds with the active sites of the UV resin, including ester carbonyls and ether oxygens. If hydrogen bonding occurs, the O–H/N–H stretching vibration frequencies would decrease, which is qualitatively consistent with the observed redshift. This is also in agreement with the experimental finding that the GA-MC coating exhibits superior adhesion compared to the SA-MC coating, as stronger interfacial bonding would be expected to enhance the adhesion strength between the coating and the substrate. On the other hand, these natural polymer materials are inherently hygroscopic, and adsorbed water also shows a broad OH stretching vibration peak centered around ~3400 cm^−1^. Moreover, given that the mass fraction of microcapsules in the coating is only 9%, the contribution of the wall materials to the overall infrared spectrum is limited, and the redshift amplitude of 83 cm^−1^ (from 3464 to 3381 cm^−1^) is relatively modest; therefore, the contributions of adsorbed water, inter-sample variability, or baseline drift cannot be ruled out. In summary, while the FTIR redshift may provide indirect clues regarding interfacial interactions, FTIR spectroscopy alone cannot distinguish between the contributions of hydrogen bonding and adsorbed water, nor can it directly establish a causal relationship. This hypothesis therefore requires further verification through direct characterization techniques such as XPS, solid-state NMR, or two-dimensional infrared correlation spectroscopy.

The absorption peak at 2920 cm^−1^ is attributed to the aliphatic C–H stretching vibrations of the long-chain alkyl groups of acrylate monomers in the UV resin [45]. The strong peak at 1720 cm^−1^ corresponded to the ester carbonyl (C=O) stretching vibration, which is a typical characteristic of UV polyurethane acrylate resin. The absorption peak at 1510 cm^−1^ is assigned to the aromatic ring skeleton vibration of SA [46], which was only identifiable in the SA-MC coating; neither the GA-MC coating nor the blank coating showed obvious absorption at this position, making it a marker peak for the presence of SA-MCs. The absorption peaks at 1460 cm^−1^ and 1413 cm^−1^ are attributed to C–H bending vibrations. The absorption peak at 1040 cm^−1^ was related to the C–O–C stretching vibrations in the CS skeleton and GA; compared with the blank coating, no obvious shift in this peak position was observed after the addition of GA-MCs, but the peak shape was relatively sharper, which may be related to the more uniform dispersion of GA-MCs in the coating and the locally higher microcapsule concentration. The absorption peak at 808 cm^−1^ is attributed to the out-of-plane C–H bending vibration of the aromatic ring in the CVL molecule, which was weakly manifested in both types of microcapsule coatings.

It is noteworthy that the characteristic absorption bands of the microcapsule wall and core materials (such as the C–O–C of CS/GA and the aromatic ring vibrations of CVL) were relatively weak in the coating spectra. The main reasons included: (1) the microcapsule addition amount was only 9%, so the mass proportion of wall and core materials in the overall coating was limited; (2) the microcapsules were encapsulated by the UV resin matrix, and the FTIR signal was masked by the strong absorption of the resin. Therefore, judging the strength of interfacial bonding solely based on peak intensity changes was insufficient.

### 3.2. Color-Changing Performance Analysis

**Figure 8:**
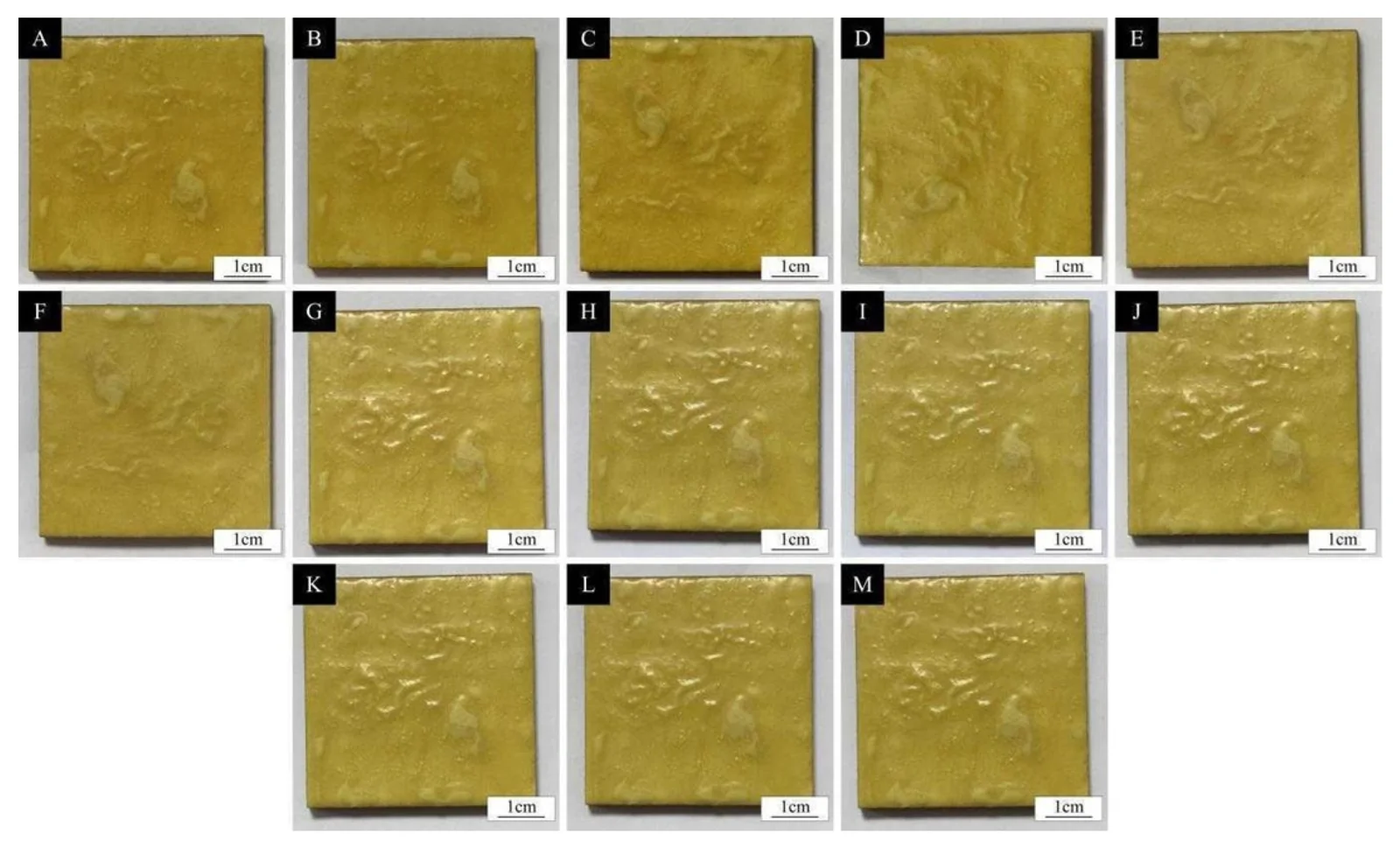
Color change of #5 coating with increasing temperature: (**A**) −10 °C, (**B**) −5 °C, (**C**) 0 °C, (**D**) 5 °C, (**E**) 10 °C, (**F**) 15 °C, (**G**) 20 °C, (**H**) 25 °C, (**I**) 30 °C, (**J**) 35 °C, (**K**) 40 °C, (**L**) 45 °C, (**M**) 50 °C.

Table S4 shows the colorimetric parameters and Δ*E* changes of the UV coatings on the basswood surface with SA-MCs added at different temperatures. Overall, as the temperature gradually increased from −10 °C to 50 °C, the Δ*E* of all samples showed an increasing trend, indicating that the coatings exhibited thermochromic response throughout the entire temperature range. The Δ*E* values of the control group without microcapsules were relatively low, indicating that the pure UV coating itself had almost no temperature-responsive capability. With the increase in microcapsule mass fraction, the temperature-responsive capability of the coatings was enhanced. The Δ*E* values of Samples #1 and #2 reached 5.0 and 5.1 at 50 °C, respectively. Sample #5 with 9% microcapsule addition reached a Δ*E* of 7.2 at 50 °C, showing a clear thermochromic response. It should be noted that although the Δ*E* value of Sample #5 exceeds the industrial threshold of Δ*E* > 5 for a perceptible color difference, its visual transition from light yellow to yellow-green remains relatively moderate. This is not contradictory: the Δ*E* is primarily contributed by a 7.4 increase in the b* value (yellow axis), with only a ~2.0 change in lightness, representing a shift within the same hue family rather than across different hue families, thus producing a weaker visual impact. Additionally, human eyes are more sensitive to hue and saturation changes in the blue-purple region than in the yellow region. Therefore, the calculated Δ*E* and the visual records in Figure 8 are both accurate and logically consistent.

Table S5 shows the colorimetric parameters and Δ*E* changes of the UV coatings on the basswood surface with GA-MCs added at different temperatures. As the microcapsule mass fraction increased from 1% to 9%, the Δ*E* values of the coatings increased significantly. Sample #6 reached a Δ*E* of 15.1 at 50 °C, while Samples #7, #8, #9, and #10 reached 24.7, 32.2, 40.0, and 42.4, respectively, showing a gradual increasing trend. This indicated that the increase in microcapsule content could significantly enhance the color development amplitude and temperature sensitivity of the coatings. Most samples showed a sudden increase in Δ*E* at around 20 °C, indicating that this temperature range was the main response interval for the thermochromic reaction of the microcapsules. As the temperature further increased, the Δ*E* values continued to increase but the rate of change gradually slowed down, indicating that the color development reaction was gradually approaching completion.

As the microcapsule mass fraction increased, the thermochromic performance of the UV coatings on the basswood surface was significantly enhanced. Both types of coatings with 9% microcapsule addition exhibited better Δ*E* and temperature-responsive capability, providing a reliable performance basis for practical applications. Further heating-cooling cycle experiments were conducted to observe the color-changing performance of the coatings on the basswood surface as the temperature decreased. Tables S6 and S7 show the colorimetric parameters and Δ*E* changes of the two thermochromic microcapsule coatings during the cooling process. The results showed that the coating color reversibly recovered upon cooling to the low-temperature state, and the Δ*E* gradually decreased with cooling, reflecting good reversible color-changing performance and cycle stability. Both types of coatings with microcapsules exhibited good cyclic response capability during both heating and cooling processes, indicating that the microcapsule structures were stable and could achieve relatively reliable reversible thermochromic effects.

**Figure 9:**
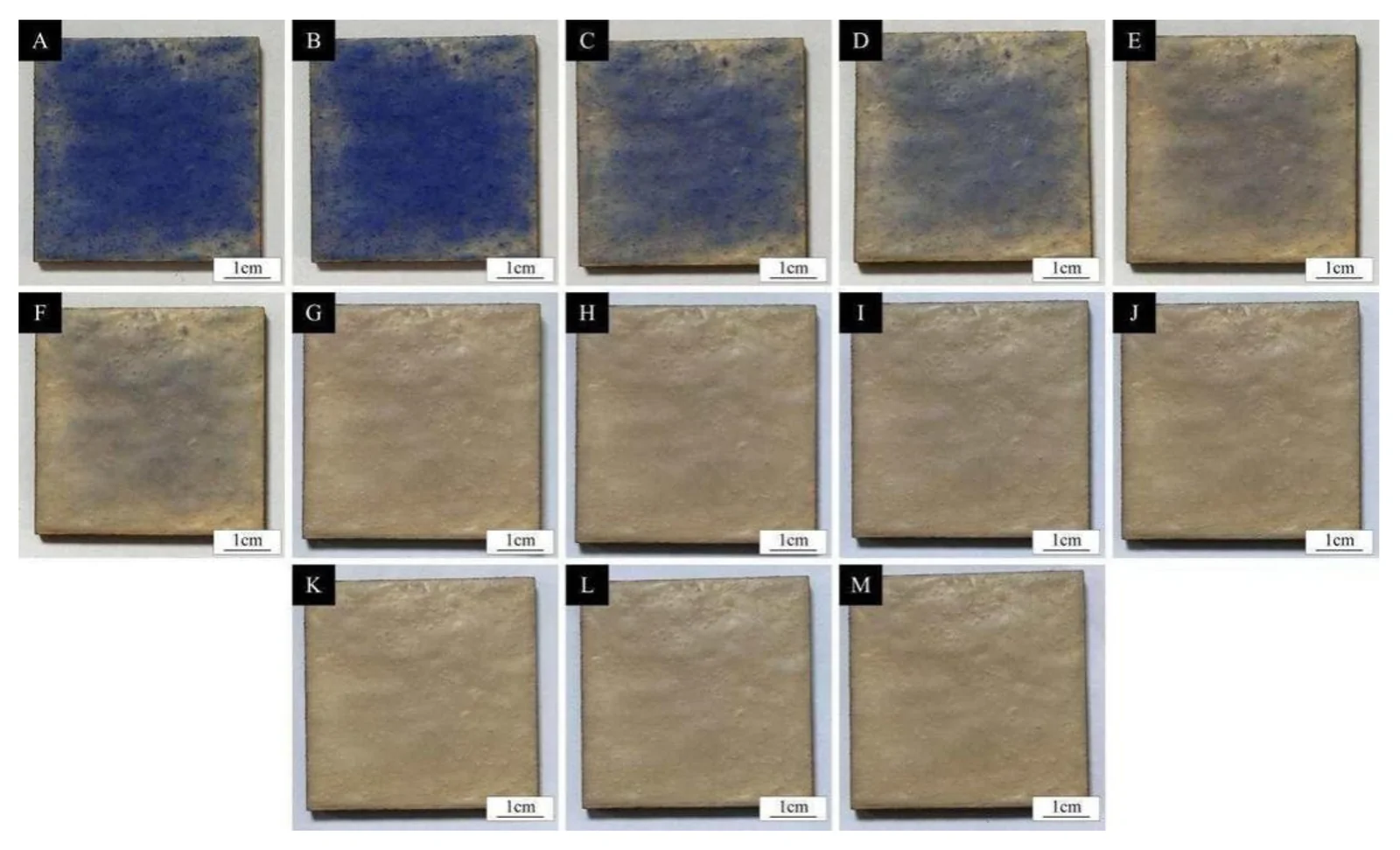
Color change of #10 coating with increasing temperature: (**A**) −10 °C, (**B**) −5 °C, (**C**) 0 °C, (**D**) 5 °C, (**E**) 10 °C, (**F**) 15 °C, (**G**) 20 °C, (**H**) 25 °C, (**I**) 30 °C, (**J**) 35 °C, (**K**) 40 °C, (**L**) 45 °C, (**M**) 50 °C.

Figure 8 and Figure 9 show the macroscopic color-changing effects of the coatings on the basswood surface with temperature. Both types of coatings with microcapsules exhibited typical reversible thermochromic characteristics, and the color-changing behavior showed a clear correspondence with temperature changes. Sample #5 with 9% SA-MCs showed a light yellow color in the low-temperature range of −10~10 °C. As the temperature increased to 15~25 °C, the yellow tone gradually deepened. When the temperature further rose to 30~50 °C, the coating color continued to deepen toward a warm yellow, and the color saturation and surface uniformity remained good, showing a mild and broad gradual change pattern, reflecting a warm-tone gradual change trend that intensified with rising temperature. It could be observed that the surface of Sample #5 had relatively obvious unevenness, and some degree of particle aggregation was observed in local areas, which might be related to the relatively large particle size of SA-MCs and their poor dispersion uniformity in the coating. From the variation pattern of CIELab parameters, the *L* value showed a continuous increasing trend with rising temperature, indicating that the overall brightness of the coating gradually increased. This phenomenon was consistent with the visual brightness enhancement caused by the strengthening of the yellow component. The *b* value showed a continuous increasing trend, indicating that the yellow-axis component was continuously enhanced, which was the dominant factor driving the macroscopic transition from light yellow to warm yellow.

Sample #10 with 9% GA-MCs exhibited a more significant color-changing effect. In the low-temperature range of −10~0 °C, the coating maintained a highly saturated blue color with uniform color distribution and obvious contrast. As the temperature increased to 5–15 °C, the coating entered a rapid color-changing transition stage, in which the blue component significantly weakened, and the coating gradually shifted from blue to light blue and light gray, with obvious color contrast. In the high-temperature range of 20–50 °C, the blue color basically faded, and the coating finally appeared as a uniform light gray, showing a clear reversible color-changing path from highly saturated blue to low-saturation blue and light gray. Compared with Sample #5, Sample #10 had better surface smoothness and higher macroscopic color-changing uniformity. From the CIELab parameter changes, it could be further observed that the *L* value of Sample #10 showed a significant increasing trend during the heating process, indicating that the brightness of the system continuously increased, corresponding to the macroscopic transition from dark blue at low temperature to a light-colored state. The *a* value gradually changed from near zero or slightly negative at low temperature to positive and tended to stabilize, indicating that the blue-dominant effect gradually weakened and the overall hue shifted toward neutral colors. The *b* value rapidly increased from negative at low temperature to positive, indicating that the system gradually transitioned from a cool blue tone to a gray-white tone. At the same time, the Δ*E* value increased most significantly in the temperature range of 5–20 °C, indicating that this temperature range was the main color-changing response interval of the system, in which the blue component decayed relatively fast, and the color change was sensitive with obvious visual contrast. After 20 °C, the changes in each colorimetric parameter gradually leveled off, indicating that the GA-MC microcapsule had basically completed the transition from the colored state to the decolored state, and the coating finally presented a stable and uniform light gray appearance. Overall, the variation pattern of CIELab parameters was consistent with the macroscopic observation results, further verifying that Sample #10 had good temperature responsiveness and comprehensive hue change characteristics.

**Figure 10:**
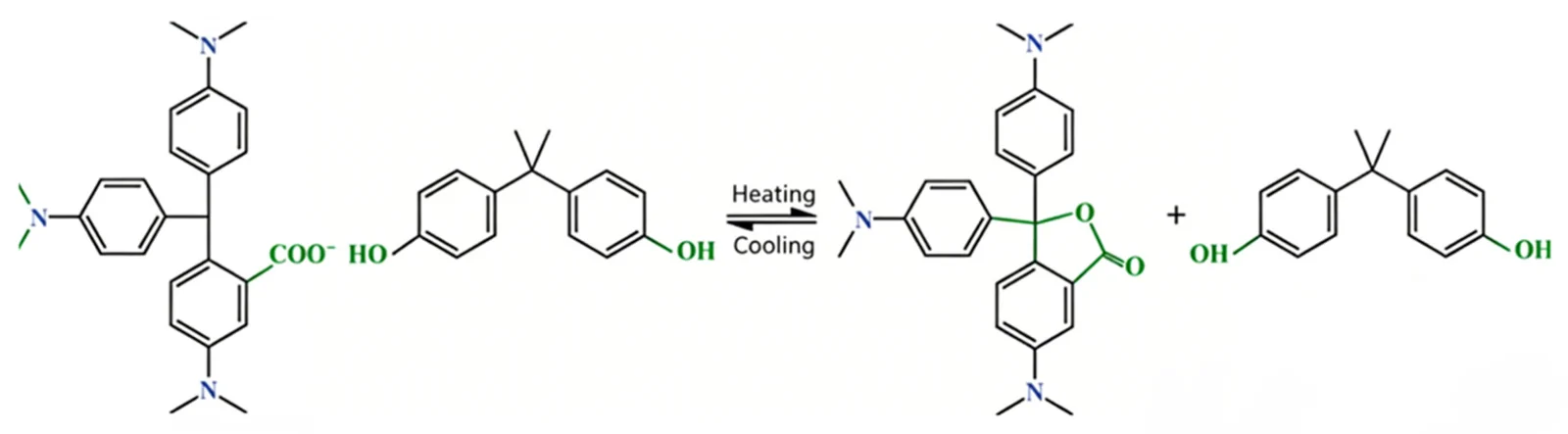
Reversible thermochromic mechanism of the CVL–BPA system.

The reversible thermochromic mechanism of the CVL-BPA system is illustrated in Figure 10. The temperature-sensitive color-changing behavior of the CS-SA encapsulated thermochromic microcapsules mainly originated from the reversible color-developing and fading mechanism between the leuco dye CVL and the color developer BPA, which was based on molecular configuration changes. At low temperatures, the solvent DA was in a solid or highly viscous state, and molecular motion was restricted. Under the induction of BPA, the lactone ring of CVL tended to undergo ring-opening reaction, forming an open-ring structure with an extended conjugated system, which shifted the absorption spectrum from the UV region to the visible region, and the system exhibited a colored state. As the temperature increased, DA gradually melted, molecular motion in the system was enhanced, and the open-ring structure of CVL converted back to the thermodynamically more stable closed-ring form. The conjugated system was disrupted, the color-developing degree gradually weakened, and the color of the system changed accordingly. When the temperature exceeded the critical color-changing interval, CVL mainly existed in the closed-ring structure, and the color of the system remained stable.

### 3.3. Optical Performance Analysis

Table S8 shows the changes in gloss and reflectance of the coating films on the basswood surface with different types and contents of microcapsules. As the microcapsule addition increased, the gloss of the coating films at incident angles of 20°, 60°, and 85° all showed a significant decreasing trend, while the 60° gloss loss rate continuously increased, indicating that the introduction of microcapsules reduced the gloss performance of the coating surface. The control sample without microcapsules had a gloss of 24.20 GU at 60° incident angle, showing relatively high surface reflectance. When SA-MCs were added, with the content increasing from 1% to 9%, the 60° gloss gradually decreased from 20.60 GU to 10.60 GU, while the 60° gloss loss rate increased from 14.88% to 56.20%. Combined with the SEM images, as the SA-MC addition increased, the number of particles on the coating surface gradually increased, the surface unevenness became more obvious, and the decreased smoothness enhanced light scattering and weakened specular reflection, reducing gloss. In contrast, the GA-MC coatings exhibited a more significant gloss reduction. Under the same content conditions, the gloss of this system at 60° incident angle was significantly lower than that of the SA-MC system. For example, at 9% addition, the gloss was 4.70 GU, and the 60° gloss loss rate reached 80.58%. Combined with the SEM results, GA-MCs had relatively smaller particle sizes and denser distribution, which more easily formed a dense particle accumulation structure on the coating surface at high addition levels, further increasing the surface microscopic roughness. The incident light underwent stronger diffuse reflection and scattering, thereby significantly weakening the specular reflection, and therefore the gloss reduction was more obvious.

**Figure 11:**
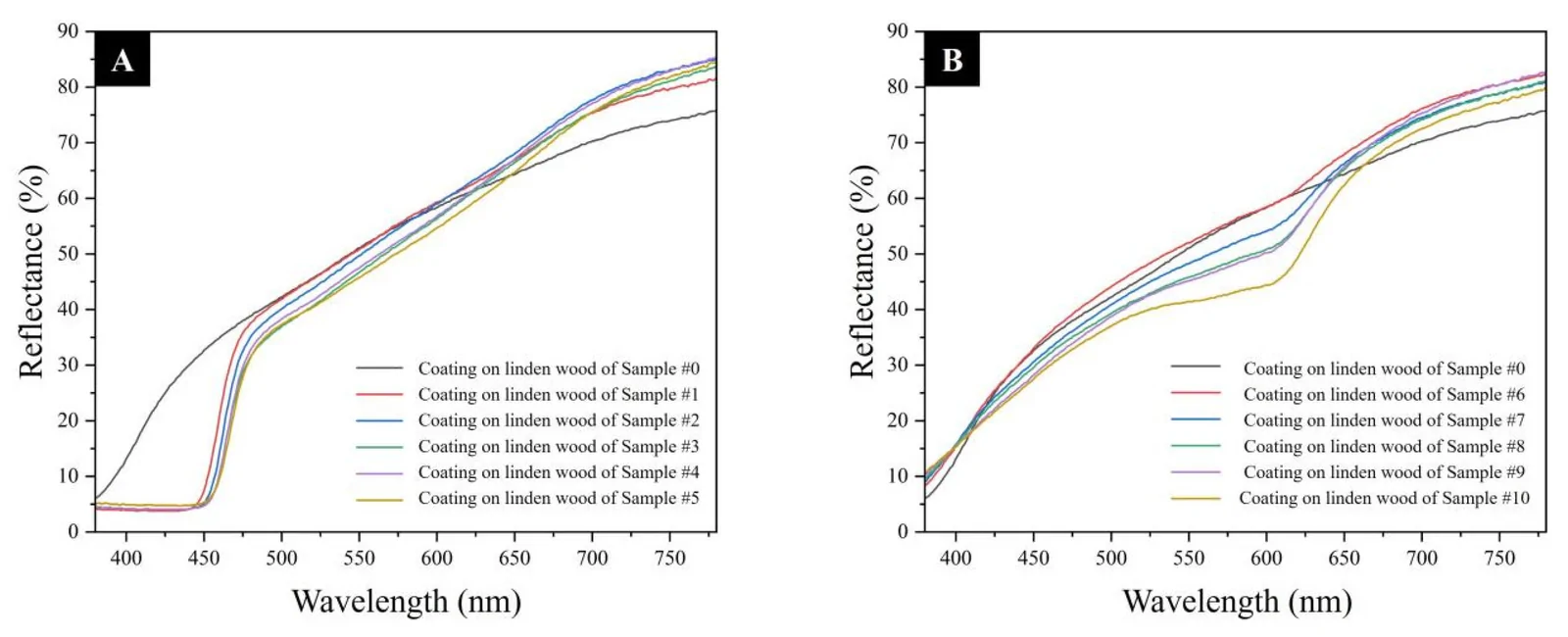
Reflectivity of surface coatings on basswood: (**A**) coatings containing SA-MCs, (**B**) coatings containing GA-MCs.

Figure 11 shows the reflectance spectra of the coatings on the basswood surface with different types of thermochromic microcapsules. The reflectance of the control sample without microcapsules was 51.65%. When SA-MCs were added, the overall reflectance of the coatings slightly decreased, and showed a small fluctuating downward trend with increasing microcapsule content, gradually decreasing from 49.88% to 47.66%. From the spectral curves, it could be observed that the samples with SA-MCs showed obvious low-reflectance broadband absorption characteristics in the violet-blue region of 400–450 nm, with reflectance generally below 5%, and a turning point of rapid increase in reflectance appeared near the blue-cyan region of 450–500 nm, indicating that this band was the main absorption band of the system. Since short-wavelength blue light was significantly absorbed, while green, yellow, orange, and red light above 500 nm could be relatively reflected, the coatings macroscopically exhibited a light yellow to yellow-green hue. This might be attributed to the fact that the SA-MC system exhibited a light yellow to yellow-green hue in the coating, which was relatively close to the natural light yellow background of the basswood substrate. At the same time, the introduction of microcapsule particles increased the microscopic roughness of the coating surface, causing more scattering of incident light at the interface, thereby reducing the overall reflectance to a certain extent.

For the coatings with GA-MCs, the reflectance was higher than that of the control sample at low addition levels. For example, Sample #6 reached a reflectance of 54.35%. From the spectral characteristics, this group of samples also showed obvious absorption in the violet-blue-green region of 400–500 nm, but the absorption intensity was generally weaker than that of the SA-MC system, and the reflectance increased rapidly after about 550 nm, indicating that this system mainly reflected yellow, orange, and red light bands. This was mainly related to the dark blue color of the microcapsules at low temperature. The darker color formed a clear contrast with the light background of the basswood, and the dispersion of microcapsule particles in the coating caused multiple scattering and reflection of some light, thereby increasing the measured apparent reflectance. As the GA-MC microcapsule content continued to increase, the reflectance gradually decreased to 48.10%. This indicated that high-content microcapsules formed a denser particle structure on the coating surface, enhancing light scattering and absorption, reducing the effective reflected luminous flux, and thus causing the decrease in reflectance. In the spectral curve of Sample #10 with high content, an obvious inflection point of reflectance change appeared near the yellow region of about 550–600 nm, indicating that the light absorption characteristics in this band had changed, and the overall absorption in the range of 400–600 nm was enhanced, with only relatively reflection of long-wavelength light above 600 nm. This was also an important reason for the overall decrease in reflectance of the system.

### 3.4. Roughness and Mechanical Performance Analysis

Table S9 shows the roughness and mechanical performance test results of the UV coatings on the basswood surface with different thermochromic microcapsules. The roughness of the control sample without microcapsules was 0.279 μm, indicating that the surface of the pure UV coating was relatively smooth. When thermochromic microcapsules were introduced, the roughness of both types of coatings increased significantly and showed a continuous upward trend with increasing microcapsule addition. This indicated that as the microcapsule content increased, some microcapsules formed microscopic protruding structures during the curing process, gradually transforming the coating film surface from relatively smooth to a microstructure with obvious particle characteristics. Combined with the trend of gloss changes, it could be observed that as roughness increased, the gloss of the coatings gradually decreased, while the 60° gloss loss rate increased significantly. This is because the rough surface enhanced the diffuse reflection and scattering of incident light at the coating interface, thereby weakening the specular reflection capability and resulting in decreased gloss. It could be seen that the introduction of microcapsules and the increase in their content not only changed the microstructure of the coatings, but also directly affected the optical properties, gradually transforming the coating film from a relatively smooth surface to a low-gloss, relatively rough surface morphology.

The control sample without microcapsules had a hardness of 6H, an impact resistance grade of 4, and an adhesion grade of 1, indicating that the UV scratch-resistant topcoat formed a dense film structure with high surface hardness and good bonding performance with the substrate under unmodified conditions. When SA-MCs were introduced, at low addition levels, the hardness of Samples #1 and #2 remained at 6H, and the impact resistance was basically maintained at Grade 4, indicating that a small amount of microcapsules had little effect on the overall structure of the UV coating film. However, as the microcapsule addition increased, the impact resistance grade gradually decreased from Grade 4 to Grade 3, while the adhesion grade gradually deteriorated from Grade 1 to Grade 4, indicating that the bonding performance at the coating-substrate interface was somewhat weakened. When the addition further increased to Sample #5, the coating hardness decreased from 6H to 5H. This is mainly because the increased content of microcapsule particles in the coating formed more interface defects and microscopic voids in the curing network, weakening the continuity of the UV resin crosslinked structure, thereby reducing the overall mechanical strength of the coating film. At the same time, the enrichment of microcapsule particles at the interface might also weaken the mechanical interlocking between the coating and the basswood substrate, resulting in decreased adhesion.

The coating hardness of the GA-MC system was generally slightly lower than that of the SA-MC system. In the low-to-medium addition range, the hardness of Samples #6 to #8 remained at 5H, the impact resistance grade remained at Grade 4, and the adhesion remained at Grade 1, indicating that this type of microcapsule caused less damage to the coating film structure at appropriate addition levels, and the coatings could still maintain good mechanical stability. When the microcapsule content further increased to Samples #9 and #10, the impact resistance grade decreased to Grade 3, the hardness further decreased to 4 H, and the adhesion grade increased to Grade 2. This indicated that higher content of microcapsules formed more particle interfaces inside the coating film, causing some disturbance to the cured UV resin network structure, thereby resulting in a certain decrease in the hardness and impact resistance of the coatings.

It is noteworthy that the GA-MC coating exhibited superior adhesion but lower hardness compared to the SA-MC coating. This apparent trade-off may be related to the chemical nature of the two wall materials. GA-MCs contain abundant polar groups, which are likely to promote interfacial adhesion with the UV resin but may simultaneously interfere with its crosslinking polymerization, potentially reducing bulk hardness. In contrast, the Schiff-base-crosslinked SA-MC wall material has fewer free polar groups, which may cause less disruption to the resin network and thus contribute to maintaining higher hardness, albeit at the expense of weaker interfacial bonding. This inverse relationship suggests a possible trade-off between adhesion and hardness in microcapsule-modified UV coatings.

Overall, as the microcapsule addition increased, the mechanical properties of the coatings in both systems showed a certain degree of decreasing trend. However, within the appropriate addition range, the coatings could still maintain high hardness and good impact resistance.

### 3.5. Short-Term Photo-Stability Analysis

Four groups of samples (4#, 5#, 9#, and 10#) with better color-changing performance were selected for a preliminary UV aging screening. Table S10 shows the chromatic values and Δ*E* of the coatings after aging as the temperature increased, reflecting the early-stage photodegradation response of the two microcapsule systems. After UV aging, for Samples 4# and 5# with SA-MCs, the Δ*E* showed a gradual increasing trend with rising temperature, reaching 4.5 and 5.2 at 50 °C, respectively, indicating that the coatings still maintained the gradual change characteristic from light yellow to yellow-green after aging. However, the overall Δ*E* was somewhat weakened compared with that before aging, which might be related to the photo-oxidation effect of UV radiation on the microcapsule wall materials and the color-developing components in the thermochromic system, thereby reducing the color response intensity. For Samples 9# and 10# with GA-MCs, the Δ*E* increased more significantly during the heating process, showing a sharp increase at around 20 °C, and reaching 29.3 and 32.8 at the high-temperature stage, respectively, indicating that this system still maintained a relatively obvious color-changing process from dark blue at low temperature to light gray at high temperature after aging. Overall, UV aging had a certain effect on the color-changing capability of both types of microcapsule coatings, but the coatings could still maintain considerable temperature-responsive characteristics, indicating that the microcapsules had certain short-term UV stability in the UV coating system.

**Figure 12:**
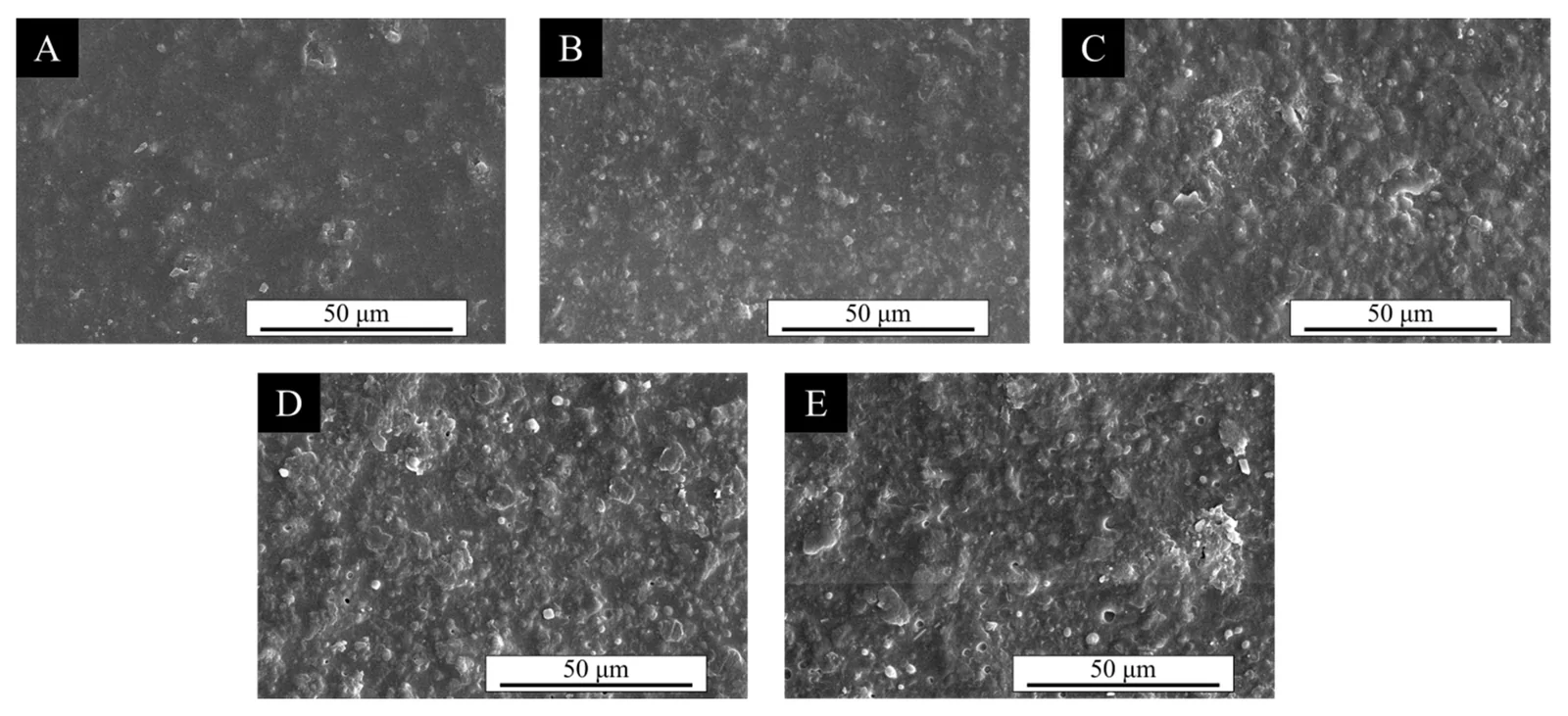
SEM images of surface coatings on basswood after 24 h accelerated UV aging: (**A**) 0#, (**B**) 4#, (**C**) 5#, (**D**) 9# (**E**) 10#.

Table S11 lists the gloss changes of each coating after UV aging, and Figure 12 shows the surface SEM morphologies of 0#, 4#, 5#, 9# and 10# coatings after 24 h accelerated UV aging. For sample 0#, the blank without microcapsules, its 60° gloss slightly decreased from 24.20 GU to 23.80 GU with minor attenuation, indicating that the photo-oxidation of the resin matrix itself was limited. After the addition of microcapsules, the gloss of coatings decreased significantly after aging, suggesting that the significant gloss reduction originated primarily from the degradation of microcapsules and their interfaces with the resin, rather than from the resin matrix. For SA-MCs-modified samples 4# and 5#, their 60° gloss dropped to 11.70 GU and 8.10 GU after aging, with corresponding gloss loss rates of 50.84% and 65.97%. Severe particle agglomeration can be observed for this system in Figure 12; numerous interfacial-debonding pits and radial microcracks appeared after aging, and the surface roughness increased sharply. The GA-MC system presented more severe gloss loss. After aging, the gloss of samples 9# and 10# was only 3.80 GU and 3.50 GU, and their gloss loss rates reached up to 84.03% and 85.29%. Although no long penetrating cracks were found on GA-MC coatings in Figure 12, microcapsules were densely stacked in the surface layer of coatings. UV irradiation produced abundant etching micropores on particle surfaces. Such multi-micro-concave-convex structures greatly enhanced diffuse reflection of light and weakened specular reflection capacity. In conclusion, the degradation of microcapsules under UV irradiation, together with increased surface roughness, may serve as the core reasons for the remarkable gloss attenuation of the two types of modified coatings after aging, while the photo-oxidation of the resin matrix itself played a minor role.

The 24 h UV aging exposure adopted in this study was intended as an early-stage screening; therefore, it cannot be directly extrapolated to predict long-term durability. Nevertheless, it was sufficient to reveal a clear differentiation in the degradation trends and photostability between the SA-MC and GA-MC systems under the same aging conditions. For a more comprehensive evaluation of practical outdoor applications, systematic long-term accelerated aging tests or natural weathering exposures will be necessary in follow-up investigations.

### 3.6. Comparison of Coating Performance with Single Addition of Two Types of Microcapsules

Table 2 compares the comprehensive performance of the coatings on the basswood surface with the single addition of the two types of thermochromic microcapsules. In terms of color-changing performance, the maximum Δ*E* of Sample #10 was 42.4, significantly higher than the 7.2 of Sample #5, indicating that GA-MCs could produce a more obvious color response during temperature change and had stronger thermochromic visual effects. This was mainly related to the color-developing characteristics of the two types of microcapsules: GA-MCs exhibited a darker blue tone at low temperature and gradually faded to nearly colorless upon heating, with a larger color contrast, thus showing higher Δ*E* values, while the color change of SA-MCs mainly manifested as a gradual transition from light yellow to yellow-green, with a relatively smaller overall Δ*E* variation. In addition, the narrow particle size distribution and uniform particle size of GA-MCs contribute to more homogeneous dispersion within the coating, which also facilitates consistency in the colorimetric response.

In terms of optical performance, the 60° gloss loss rate of Sample #5 was 56.20%, lower than the 80.58% of Sample #10, indicating that the coatings with GA-MCs more easily produced obvious gloss loss. This is mainly attributed to the narrow particle size distribution and uniform particle size of GA-MCs, which facilitated the formation of a dense and uniform particle packing layer on the coating surface. Upon incorporation, this layer enhanced light scattering at the coating surface, thereby reducing its specular reflectance. The reflectance of the two types of coatings was 47.66% and 48.10%, respectively, with little overall difference, indicating that the microcapsule type had a relatively limited effect on the overall reflectance.

Combined with roughness and mechanical properties, the roughness of Sample #5 was 0.883 μm, higher than the 0.762 μm of Sample #10, indicating that SA-MCs more easily formed obvious particle undulating structures on the coating surface at higher addition levels, thereby increasing surface roughness and further affecting adhesion performance. The hardness of Sample #5 was 5 H, higher than the 4 H of Sample #10, indicating that the SA-MC system caused relatively less damage to the crosslinked structure of the UV coating, allowing the coating film to maintain relatively high surface hardness. The impact resistance grades of both coatings were rated as level 3, indicating that their impact resistance was essentially comparable. However, in terms of adhesion, sample 10# exhibited a grade of 2, which was superior to the grade 4 of sample 5#. This was probably associated with the abundant polar groups (–OH, –COO^−^, –NH_3_^+^) in the GA-MC wall material. The red shift at 3381 cm^−1^ in the FTIR spectrum is consistent with the possible presence of hydrogen-bonding interactions. However, as FTIR provides only indirect evidence, the relationship between spectral features and adhesion performance remains to be further clarified. In addition, the narrow particle size distribution and uniform particle dispersion of GA-MCs reduced interfacial defects and stress concentration, thereby contributing to the maintained adhesion performance between the coating and the basswood substrate.

In the comparative UV-aging screening (24 h), the aged Sample #5 reached a Δ*E* of 5.2 at 50 °C, whereas the aged Sample #10 exhibited a substantially higher maximum Δ*E* of 32.8 at 35 °C, indicating that the GA-MC system retained a more pronounced thermochromic response under the tested short-term aging conditions. It should be emphasized that this 24 h UV exposure was designed as an early-stage accelerated screening test; while it was sufficient to reveal clear differences in the initial degradation trends between the two microcapsule systems, it does not represent their long-term weathering performance. In terms of gloss, both coatings suffered significant reduction after aging, with gloss loss rates of 65.97% for Sample #5 and 85.29% for Sample #10. This indicates that the GA-MC system, despite its superior color-changing retention, exhibited a more severe deterioration in surface optical stability under the current aging conditions.

Overall, Sample #10 had a larger color-changing amplitude and more obvious thermochromic response, and could still maintain strong color-changing capability after aging. However, its gloss stability was poor, and the gloss loss phenomenon was more obvious. Sample #5, although having a smaller color-changing amplitude, had better gloss retention and surface hardness, with higher overall stability. Therefore, from the perspective of thermochromic visual effects, Sample #10 had more advantages, while from the perspective of comprehensive coating stability, Sample #5 showed a more balanced performance. The two exhibited different performance priorities between thermochromic effects and comprehensive coating properties.

## 4. Conclusions

Based on the above microscopic characterization and performance testing results, this study systematically compared the comprehensive performance differences of basswood UV coatings modified with different dosages of SA-MCs and GA-MCs. With increasing microcapsule content, the color-changing amplitude of both coatings increased, while gloss and hardness exhibited a decreasing trend; within the tested range, 9% was identified as the optimal addition level for thermochromic performance. At this addition level, the SA-MC coating had an average particle size of 1.745 μm with a relatively broad distribution, a coating thickness of approximately 0.40 mm, a maximum Δ*E* of 7.2 at 50 °C (light yellow to yellow-green), a 60° gloss loss rate of 56.20%, a roughness of 0.883 μm, a pencil hardness of 5H, an impact resistance of Grade 3, and an adhesion of Grade 4. The GA-MC coating exhibited an average particle size of 1.655 μm with a narrower distribution, a coating thickness of approximately 0.27 mm, a maximum Δ*E* of 42.4 (deep blue to light gray), a 60° gloss loss rate of 80.58%, a roughness of 0.762 μm, a pencil hardness of 4H, an impact resistance of Grade 3, and an adhesion of Grade 2, with a crack-free and uniformly dispersed cross-section. Both systems demonstrated good reversible cycling stability. After short-term UV aging (24 h), the GA-MC coating still maintained a thermochromic response of Δ*E* = 32.8, outperforming the SA-MC coating (Δ*E* = 5.2), but suffered more severe gloss loss (85.29% vs. 65.97%). In summary, the GA-MC coating at 9% addition exhibits the most outstanding color-changing amplitude and adhesion, along with superior short-term photostability, making it suitable for high-visual-contrast applications; the SA-MC coating offers advantages in hardness and gloss retention, with a more balanced overall stability. This study compares the application differences between Schiff base crosslinking and electrostatic complex coacervation encapsulation strategies in wood coatings, providing an experimental basis for the selection of environmentally friendly thermochromic microcapsules. It should be noted that the 24 h UV aging test was designed only as an early-stage screening and does not represent long-term durability; subsequent work is required to characterize the interfacial chemical states via XPS and to conduct extended aging tests to support practical engineering applications.

## Figures and Tables

**Table 1:** Addition amounts of two types of microcapsules and coatings.

Sample (#)	Two-Primer–Two-Topcoat Coating Process	Microcapsule Addition Amount (%)	Microcapsule Mass (g)	UV Coating Mass per Layer (g)
0	Without microcapsules	0	0.000	0.500
1	SA-MCs added to both primer and topcoat	1	0.005	0.495
2		3	0.015	0.485
3		5	0.025	0.475
4		7	0.035	0.465
5		9	0.045	0.455
6	GA-MCs added to both primer and topcoat	1	0.005	0.495
7		3	0.015	0.485
8		5	0.025	0.475
9		7	0.035	0.465
10		9	0.045	0.455

**Table 2:** Comparison of surface coating properties of two thermochromic microcapsules added individually for basswood.

Coating (#)	Color-Changing Performance	Optical Performance		Roughness and Mechanical Properties				Short-Term Photo-Stability	
	Maximum Δ*E*	Gloss Loss Rate (%)	Reflectance (%)	Roughness (μm)	Hardness (H)	Impact Resistance Grade	Adhesion Grade	Maximum Δ*E*	Gloss Loss Rate (%)
5	7.2	56.20	47.66	0.883	5	3	4	5.2	65.97
10	42.4	80.58	48.10	0.762	4	3	2	32.8	85.29

## Data Availability

The authors confirm that the data supporting the findings of this study are available within the article.

## Supplementary Materials

The following supporting information can be downloaded at: https://www.mdpi.com/article/10.3390/polym18162017/s1, Table S1: Experimental materials; Table S2: Experimental equipments; Table S3: Impact site grade; Table S4: Chromaticity and Δ*E* of SA-MCs coatings with increasing temperature; Table S5: Chromaticity and Δ*E* of GA-MCs coatings with increasing temperature; Table S6: Chromaticity and Δ*E* of SA-MCs microcapsule coatings with decreasing temperature; Table S7: Chromaticity and Δ*E* of GA-MCs microcapsule coatings with decreasing temperature; Table S8: Coatings’ surface glossiness and reflectivity with different microcapsules; Table S9: Roughness and mechanical properties of basswood coatings’ surface with different microcapsules; Table S10: Chromaticity and Δ*E* of aged coatings with increasing temperature; Table S11: Aged coatings’ surface glossiness.
