# Supplementary material for: Effects of Syringaldehyde/Gum Arabic Composite Chitosan Thermochromic Microcapsules on the Coating Properties of Basswood Surface
Source: Polymers (Basel). 2026 Aug 20;18(16):2017. doi: 10.3390/polym18162017 (PMC13517314; doi:10.3390/polym18162017)
Supplement: Supplementary file 1 [file polymers-18-02017-s001.zip › polymers-4444266-supplementary.pdf]

Table S1. Experimental materials

Experimental materials	Molecular formula	CAS No.	Manufacturer
Crystal violet lactone (CVL)	$C_{26}H_{29}N_3O_2$	1552-42-7	Shanghai Haiyu Chemical Co., Ltd.
Bisphenol A (BPA)	$C_{15}H_{16}O_2$	80-05-7	Shanghai Aibi Chemical Reagent Co., Ltd.
Decyl alcohol	$C_{10}H_{22}O$	112-30-1	Shanghai Macklin Biochemical Co., Ltd.
Chitosan (CS)	$(C_6H_{11}NO_4)_n$	9012-76-4	Sinopharm Chemical Reagent Co., Ltd., Shanghai
Acetic acid	$C_2H_4O_2$	64-19-7	Shanghai Zhanyun Chemical Co., Ltd.
Syringaldehyde (SA)	$C_9H_{10}O_4$	134-96-3	Shanghai Dibai Biotechnology Co., Ltd.
Tween 80	$C_{185}H_{368}O_{86}$	9005-65-6	Fuzhou Feijing Biotechnology Co., Ltd.
Span 80	$C_{24}H_{44}O_6$	1338-43-8	Shandong Yousuo Chemical Technology Co., Ltd.
Lauryl alcohol (LA)	$C_{12}H_{26}O$	112-53-8	Tianjin Huasheng Chemical Reagent Co., Ltd.
Gum Arabic (GA)	$(C_6H_{10}O_5)_n$	9000-01-5	Tianjin Zhonglian Chemical Reagent Co., Ltd.

Table S2. Experimental equipments

Experimental equipment	Model	Manufacturer
Digital thermostatic water bath	DF-101S	Muyang hongguanriyi e-commerce co., ltd.
Electronic balance	JCS-W	Yongkang huanyu weighing apparatus co., ltd.
Ultrasonic emulsification disperser	BILONG-500	Shanghai bilang instrument co., ltd.
Mini spray dryer	JA-PWGZ100	Shenyang jingao instrument technology co., ltd.
Uv single-lamp curing machine	620#	Huzhou tongxu machinery equipment co., ltd.
Optical microscope (OM)	AX10	Carl zeiss ag, germany
Scanning electron microscope (SEM)	Quanta-200	Thermo fisher scientific, usa
Fourier transform infrared spectrometer (FTIR)	VERTEX 80V	Bruker corporation, germany
Spectrophotometric colorimeter	SC-10	Shanghai hechen energy technology co., ltd.
Constant temperature heating plate	BY1010	Dongguan fengxia trading co., ltd.
Handheld thermometer	2003Y	Putian hanjiang huafeng plastic co., ltd.
Gloss meter	HG268	Shenzhen 3nh technology co., ltd.
UV-vis spectrophotometer	U-3900/3900H	Hitachi instruments (suzhou) co., ltd.
Roughness tester	J8-4C	Shanghai taiming optical instrument co., ltd.
UV yellowing resistance test chamber	WJ-UV-150	Hangzhou wujia machinery co., ltd.
Pencil hardness tester	HT-6510P	Quzhou aipu measuring instrument co., ltd.
Coating impact tester	QCJ-50	Dongguan jiaxin measuring instrument co., ltd.
Coating adhesion cross-cut tester	QFH-A	Quzhou aipu measuring instrument co., ltd.

Table S3. Impact site grade

Grade	Description
1	No visible change
2	No cracks on the coating film surface, but impact marks are visible
3	Slight cracks on the coating film surface, usually 1–2 rings of circular or arc cracks
4	Moderate to severe cracks on the coating film surface, usually 3–4 rings of circular or arc cracks
5	Severe damage on the coating film surface, usually more than 5 rings of circular or arc cracks, or coating film peeling

Table S4. Chromaticity and Δ*E* of SA-MCs coatings with increasing temperature

Sample (#)	Colorimetric parameters	-10 ℃	-5 ℃	0 ℃	5 ℃	10 ℃	15 ℃	20 ℃	25 ℃	30 ℃	35 ℃	40 ℃	45 ℃	50 ℃
0	*L*	62.4	63.2	63.8	64.4	64.5	64.6	64.7	64.8	65.0	64.7	65.2	65.3	65.3
	a	3.8	3.8	3.6	3.4	3.6	3.6	3.7	3.6	3.2	3.5	3.5	3.8	3.8
	b	15.8	15.4	15.6	15.7	15.1	15.1	15.5	15.4	15.4	14.5	15.4	16.0	16.2
	Δ*E*	-	0.9	1.4	2.0	2.2	2.3	2.3	2.4	2.7	2.7	2.8	2.9	2.9
1	*L*	62.5	63.7	64.6	64.8	65.2	66.0	66.1	66.2	66.1	66.3	66.8	67.1	67.4
	a	2.2	2.4	2.1	1.9	1.8	1.8	1.8	1.9	1.5	1.6	1.8	1.7	1.9
	b	35.7	36.5	36.5	36.3	35.1	35.9	35.9	36.3	36.7	36.5	36.5	36.4	36.5
	Δ*E*	-	1.5	2.2	2.4	2.8	3.5	3.6	3.8	3.8	3.9	4.4	4.7	5.0
2	*L*	61.6	62.7	63.6	64.5	64.7	64.7	64.6	64.7	64.8	64.6	65.3	66.1	66.3
	a	3.4	2.9	2.6	2.8	2.8	2.8	2.8	2.9	2.7	2.6	2.6	2.4	2.1
	b	39.2	39.5	39.2	39.3	39.3	39.5	40.2	40.1	39.7	40.8	40.4	40.5	40.7
	Δ*E*	-	1.2	2.2	3.0	3.2	3.2	3.2	3.3	3.3	3.5	4.0	4.8	5.1
3	*L*	60.7	62.2	62.6	63.0	62.5	63.3	63.4	63.9	64.5	64.4	64.6	64.5	65.1
	a	4.7	4.2	3.9	3.8	3.3	3.7	3.6	3.5	3.3	3.0	2.8	2.2	2.4
	b	41.7	42.9	41.0	40.9	40.5	41.5	41.6	42.1	41.4	42.8	42.7	43.1	43.4
	Δ*E*	-	1.9	2.2	2.6	2.6	2.8	2.9	3.4	4.0	4.2	4.5	4.8	5.2
4	*L*	61.6	61.5	62.5	63.2	62.9	63.4	63.6	64.5	64.6	65.4	65.2	65.6	66.3
	a	4.3	4.4	3.4	3.6	3.6	3.5	3.3	3.3	3.3	3.5	2.5	2.6	2.5
	b	41.4	42.7	42.0	41.0	42.4	42.4	42.7	42.1	42.8	42.9	43.5	44.3	45.2
	Δ*E*	-	2.0	2.2	2.6	2.6	2.8	2.9	3.4	4.1	4.2	4.5	4.8	5.2
5	*L*	62.5	63.5	64.1	64.3	64.1	64.4	64.8	64.7	64.6	65.7	66.1	66.4	67.6
	a	5.5	4.5	4.3	4.0	3.6	3.6	3.7	3.9	3.8	3.5	3.4	2.6	2.4
	b	42.5	43.5	44.3	44.5	44.4	44.2	44.1	44.6	45.7	45.0	45.7	46.5	46.5
	Δ*E*	-	1.7	2.7	3.1	3.1	3.2	3.3	3.4	4.2	4.5	5.3	6.3	7.2

Table S5. Chromaticity and ΔE of GA-MCs coatings with increasing temperature

Sample (#)	Colorimetric parameters	-10 ℃	-5 ℃	0 ℃	5 ℃	10 ℃	15 ℃	20 ℃	25 ℃	30 ℃	35 ℃	40 ℃	45 ℃	50 ℃
0	L	62.4	63.2	63.8	64.4	64.5	64.6	64.7	64.8	65.0	64.7	65.2	65.3	65.3
	a	3.8	3.8	3.6	3.4	3.6	3.6	3.7	3.6	3.2	3.5	3.5	3.8	3.8
	b	15.8	15.4	15.6	15.7	15.1	15.1	15.5	15.4	15.4	14.5	15.4	16.0	16.2
	ΔE	-	0.9	1.4	2.0	2.2	2.3	2.3	2.4	2.7	2.7	2.8	2.9	2.9
6	L	58.3	58.5	61.5	61.2	61.1	61.7	65.3	65.4	65.4	65.2	66.0	66.0	65.9
	a	-0.1	-0.9	1.2	1.6	1.7	1.8	3.9	3.5	3.9	4.0	3.7	4.0	4.1
	b	4.5	5.9	6.9	8.9	9.2	9.6	16.3	16.5	16.4	16.5	16.3	16.6	16.9
	ΔE	-	1.6	4.2	5.5	5.8	6.4	14.3	14.4	14.4	14.4	14.6	14.9	15.1
7	L	50.5	49.1	51.7	54.0	55.8	56.3	62.4	62.2	62.3	62.4	62.6	62.8	62.8
	a	-1.1	-1.4	-0.7	-0.8	0.8	1.0	4.8	4.8	4.8	4.9	4.7	4.9	4.9
	b	-4.2	-3.4	-1.7	-3.4	2.9	3.5	15.1	15.5	15.6	15.7	15.8	16.2	16.4
	ΔE	-	1.6	2.8	3.6	9.1	9.9	23.4	23.7	23.8	24.0	24.1	24.6	24.7
8	L	45.4	46.9	47.8	48.9	52.4	53.6	60.7	61.0	61.0	61.1	61.2	61.5	61.5
	a	-0.8	-0.9	-0.7	-0.3	0.8	1.1	5.7	5.9	5.5	5.8	5.8	5.7	5.8
	b	-11.1	-11.0	-8.8	-6.8	-1.4	0.0	14.7	15.1	15.4	15.4	15.5	15.5	16.0
	ΔE	-	1.5	3.3	5.6	12.1	13.9	30.7	31.2	31.4	31.5	31.6	31.8	32.2
9	L	42.2	43.1	45.8	48.7	50.8	52.6	62.0	62.8	62.7	63.0	62.8	62.9	62.9
	a	-0.3	-0.4	-0.5	-0.6	-0.1	0.5	6.2	6.3	6.3	6.1	6.2	6.0	6.0
	b	-16.2	-15.7	-11.1	-8.2	-5.3	-2.7	16.3	16.8	17.1	17.0	17.2	17.4	17.5
	ΔE	-	1.0	6.2	10.3	13.9	17.1	38.6	39.5	39.7	39.7	39.8	40.0	40.0
10	L	39.5	39.5	42.8	45.9	49.3	50.2	60.4	60.4	60.6	60.5	60.9	60.8	61.0
	a	0.6	0.4	0.0	-0.1	-0.2	0.1	6.9	7.0	6.9	7.0	6.8	6.8	6.8
	b	-19.8	-18.7	-15.1	-11.4	-7.7	-6.1	14.6	15.1	15.3	15.8	15.7	16.0	16.2
	ΔE	-	1.1	5.8	10.6	15.6	17.4	40.7	41.2	41.4	41.8	41.9	42.1	42.4

Table S6. Chromaticity and ΔE of SA-MCs microcapsule coatings with decreasing temperature

Sample (#)	Colorimetric parameters	50 ℃	45 ℃	40 ℃	35 ℃	30 ℃	25 ℃	20 ℃	15 ℃	10 ℃	5 ℃	0 ℃	-5 ℃	-10 ℃
0	L	65.3	65.2	65.1	64.9	64.9	64.9	64.9	64.8	64.5	64.5	63.8	63.0	62.5
	a	4.0	3.8	4.0	3.8	3.7	3.8	4.0	3.6	3.6	3.6	3.4	4.1	3.7
	b	15.8	15.9	15.8	15.2	15.2	15.1	15.6	15.5	14.7	15.9	15.8	15.8	15.9
	ΔE	2.8	2.7	2.6	2.5	2.5	2.5	2.4	2.3	2.3	2.0	1.3	0.6	-
1	L	67.1	67.1	66.8	66.4	66.3	66.1	65.5	65.7	65.0	64.7	64.6	63.3	62.5
	a	1.8	1.8	1.9	1.9	2.0	1.6	1.8	1.9	2.1	2.0	2.2	2.5	2.2
	b	37.3	36.3	35.7	36.8	36.9	36.6	37.2	35.7	35.3	36.8	36.5	36.2	36.0
	ΔE	4.8	4.6	4.3	4.0	3.9	3.7	3.3	3.2	2.6	2.3	2.2	0.9	-
2	L	66.0	65.8	65.2	64.8	64.7	64.7	64.4	64.5	64.5	63.6	63.3	62.6	61.4

	a	2.7	2.4	2.7	2.9	2.9	3.2	1.9	2.2	2.6	2.2	2.5	2.8	3.3
	b	40.9	40.2	39.3	40.8	39.7	39.8	39.2	39.2	39.0	39.1	38.8	39.1	39.1
	Δ*E*	5.0	4.6	3.9	3.8	3.4	3.4	3.3	3.3	3.2	2.5	2.1	1.3	-
3	*L*	65.0	64.6	64.5	64.3	64.2	63.9	63.6	63.4	62.7	62.5	62.5	62.1	60.6
	a	2.2	2.3	2.2	2.7	3.3	3.4	3.3	3.7	3.4	3.5	3.9	5.0	4.8
	b	43.0	43.5	42.6	42.6	41.2	41.8	41.6	40.7	40.3	40.6	40.6	42.2	41.9
	Δ*E*	5.2	5.0	4.7	4.3	4.0	3.6	3.4	3.2	3.0	2.6	2.5	1.5	-
4	*L*	65.4	65.3	65.1	65.2	64.6	64.6	63.4	63.4	63.2	63.1	61.5	62.7	61.8
	a	2.1	2.9	2.6	3.9	3.3	3.4	3.0	3.5	3.7	3.7	4.3	3.6	4.1
	b	44.3	44.9	43.1	42.8	42.8	41.8	42.3	42.1	41.3	42	42.6	42	41.5
	Δ*E*	5.0	5.0	4.0	3.6	3.2	2.9	2.1	1.8	1.5	1.4	1.2	1.1	-
5	*L*	67.3	66.4	66.3	65.6	65.1	64.9	64.9	64.7	64.4	64.3	64.3	63.4	62.9
	a	2.6	2.4	3.4	3.5	3.7	3.6	3.7	3.8	3.7	4.0	4.1	4.4	5.5
	b	47.0	46.2	45.7	45.4	45.6	44.3	44.3	44.2	44.4	44.2	44.2	43.7	42.5
	Δ*E*	6.9	6.0	5.1	4.4	4.2	3.3	3.2	3.0	3.0	2.7	2.6	1.7	-

Table S7. Chromaticity and Δ*E* of GA-MCs microcapsule coatings with decreasing temperature

Sample (#)	Colorimetric parameters	50 ℃	45 ℃	40 ℃	35 ℃	30 ℃	25 ℃	20 ℃	15 ℃	10 ℃	5 ℃	0 ℃	-5 ℃	-10 ℃
0	*L*	65.3	65.2	65.1	64.9	64.9	64.9	64.9	64.8	64.5	64.5	63.8	63.0	62.5
	a	4.0	3.8	4.0	3.8	3.7	3.8	4.0	3.6	3.6	3.6	3.4	4.1	3.7
	b	15.8	15.9	15.8	15.2	15.2	15.1	15.6	15.5	14.7	15.9	15.8	15.8	15.9
	Δ*E*	2.8	2.7	2.6	2.5	2.5	2.5	2.4	2.3	2.3	2.0	1.3	0.6	-
6	*L*	67.2	67.1	65.9	65.6	66.5	66.0	65.4	62.4	61.5	62.3	61.8	58.3	59.6
	a	4.2	4.0	3.9	4.4	4.0	3.9	4.0	2.0	2.1	1.7	1.7	-0.8	0.0
	b	17.7	17.5	17.7	17.5	16.9	16.8	16.6	10.0	10.4	9.9	6.0	5.8	5.4
	Δ*E*	15.1	14.8	14.4	14.2	14.0	13.6	13.2	5.7	5.7	5.5	2.8	1.6	-
7	*L*	64.9	64.4	64.8	63.3	63.3	62.6	62.7	57.2	55.5	53.8	53.8	49.5	51.5
	a	5.8	5.1	5.7	5.1	5.1	5.2	4.4	1.7	1.0	-0.2	0.0	-1.1	-0.3
	b	16.9	16.7	15.4	16.3	16.3	15.9	14.4	5.2	3.2	-0.1	-0.3	-2.3	-2.2
	Δ*E*	24.1	23.5	22.9	22.6	22.6	21.9	20.6	9.6	6.8	3.1	3.0	2.2	-
8	*L*	61.3	61.1	60.8	60.4	60.2	60.2	60.1	52.9	52.4	49.0	47.0	46.1	45.4
	a	5.4	5.4	5.5	5.0	4.6	4.8	4.9	1.0	0.8	0.0	-0.7	-0.4	-0.5
	b	15.2	15.2	15.2	15.1	15.3	15.2	14.7	-0.7	-1.2	-6.1	-9.5	-10.8	-11.9
	Δ*E*	32.0	31.9	31.7	31.4	31.4	31.3	30.9	13.6	12.9	6.8	2.9	1.3	-
9	*L*	62.3	62.2	62.5	62.7	62.7	62.5	61.5	53.0	50.5	48.9	45.2	43.3	42.1
	a	6.1	6.2	6.3	6.2	6.0	6.2	6.5	0.9	-0.7	-0.6	-0.6	-0.2	-0.2
	b	17.4	17.2	17.1	17.0	16.8	16.2	16.2	-2.1	-6.2	-8.4	-13.0	-14.1	-16.5
	Δ*E*	40.0	39.8	39.8	39.8	39.6	39.1	38.6	18.1	13.3	10.6	4.7	2.7	-
10	*L*	61.1	60.6	60.7	60.6	60.4	60.3	60.2	50.2	49.2	46.5	42.7	39.1	39.5
	a	6.8	7.0	6.8	7.0	7.0	6.8	6.7	0.3	-0.5	-0.3	0.1	0.5	0.8

b	16.4	16.3	15.9	15.9	15.4	14.6	14.6	-5.2	-8.0	-10.3	-14.6	-17.4	-19.0
ΔE	41.9	41.6	41.3	41.3	40.7	40.0	39.9	17.5	14.7	11.2	5.5	1.7	-

Table S8. Coatings surface glossiness and reflectivity with different microcapsules

Sample (#)	Coating	Gloss (GU)			60° gloss loss rate (%)	Reflectance (%)
		Light incident angle 20°	Light incident angle 60°	Light incident angle 85°		
0	Without microcapsules	4.00	24.20	42.20	-	51.65
1	Basswood coating with SA-MCs	3.80	20.60	39.80	14.88	49.88
2		3.30	19.50	38.80	19.42	50.07
3		2.50	18.20	34.60	24.79	48.02
4		2.20	13.90	27.30	42.56	48.85
5		1.80	10.60	22.10	56.20	47.66
6	Basswood coating with GA-MCs	1.40	10.10	14.70	58.26	54.35
7		1.30	9.30	13.40	61.57	52.03
8		1.10	7.10	9.40	70.66	50.80
9		0.70	5.10	8.50	78.93	50.78
10		0.70	4.70	4.40	80.58	48.10

Table S9. Roughness and mechanical properties of basswood coatings surface with different microcapsules

Sample (#)	Coating	Roughness (μm)	Hardness raw data (n=3)	Hardness (H, mode)	Impact raw data (n=5)	Impact resistance grade (mode)	Adhesion grade
0	Without microcapsules	0.279	6, 6, 6	6	4, 4, 4, 4, 4	4	1
1	Basswood coating with SA-MCs	0.398	6, 6, 6	6	4, 4, 4, 4, 4	4	1
2		0.504	6, 6, 6	6	4, 4, 4, 4, 4	4	2
3		0.545	6, 6, 6	6	3, 3, 3, 3, 3	3	2
4		0.602	6, 6, 6	6	3, 3, 3, 3, 3	3	3
5		0.883	5, 5, 5	5	3, 3, 3, 3, 3	3	4
6	Basswood coating with GA-MCs	0.476	5, 5, 5	5	4, 4, 4, 4, 4	4	1
7		0.529	5, 5, 5	5	4, 4, 4, 4, 4	4	1
8		0.649	5, 5, 5	5	4, 4, 4, 4, 4	4	1
9		0.652	5, 5, 5	5	3, 3, 3, 3, 3	3	1
10		0.762	4, 4, 4	4	3, 3, 3, 3, 3	3	2

Table S10. Chromaticity and Δ*E* of aged coatings with increasing temperature

Sample (#)	Colorimetric parameters	-10 ℃	-5 ℃	0 ℃	5 ℃	10 ℃	15 ℃	20 ℃	25 ℃	30 ℃	35 ℃	40 ℃	45 ℃	50 ℃
0	*L*	63.3	63.4	63.2	63.8	62.7	64.8	64.5	64.3	64.3	63.8	64.7	63.7	64.6
	a	4.4	4.5	4.1	4.3	4.1	4.4	4.3	4.2	4.2	3.9	4.0	4.0	3.8
	b	19.2	19.0	18.6	18.1	17.8	18.4	17.7	17.5	17.5	17.0	17.2	16.6	16.9
	ΔE	-	0.2	0.7	1.2	1.6	1.7	1.9	2.0	2.0	2.3	2.5	2.7	2.7
4	*L*	62.3	63.2	63.3	63.7	64.1	63.3	63.5	64.1	63.8	62.5	63.3	62.3	62.7
	a	8.1	7.7	7.6	7.0	7.0	6.7	6.8	6.4	6.8	6.6	6.6	6.1	5.4
	b	46.4	46.9	45.6	45.9	46.4	45.1	44.5	44.5	43.9	43.0	42.8	42.6	42.8
	ΔE	-	1.1	1.4	1.8	2.1	2.2	2.6	3.1	3.2	3.7	4.0	4.3	4.5
5	*L*	62.9	62.3	62.3	63.8	63.5	61.6	62.9	62.0	61.2	60.8	61.2	60.7	60.9
	a	8.5	8.4	7.9	7.1	7.1	7.5	7.0	7.5	7.3	7.3	7.1	7.1	6.3
	b	46.8	46.3	44.6	44.6	43.9	43.7	43.6	43.5	43.0	42.7	42.4	42.4	42.5
	ΔE	-	0.8	2.4	2.8	3.3	3.5	3.5	3.6	4.3	4.8	4.9	5.1	5.2
9	*L*	45.8	47.1	53.0	56.3	59.1	61.9	61.3	61.9	60.1	60.8	61.0	60.1	60.6
	a	-0.9	-0.5	1.3	2.7	4.1	6.0	5.9	5.8	6.4	6.0	5.8	6.1	6.1
	b	-2.2	-0.5	6.8	11.6	16.2	20.5	21.1	21.0	22.0	22.1	22.8	22.4	22.1
	ΔE	-	2.2	11.7	17.7	23.2	28.7	28.8	29.0	29.0	29.4	30.0	29.3	29.3
10	*L*	41.3	43.0	47.6	53.0	55.7	59.4	58.0	59.3	59.0	59.0	58.4	58.1	58.1
	a	-0.5	-0.9	0.4	2.7	3.2	5.4	6.7	6.6	6.8	6.4	6.3	6.1	6.3
	b	-4.0	-4.9	3.7	10.4	13.6	19.3	21.8	22.0	22.2	22.7	22.5	22.5	22.4
	ΔE	-	2.0	10.0	18.8	23.0	30.1	31.6	32.4	32.5	32.8	32.3	32.1	32.0

Table S11. Aged coatings surface glossiness

Sample (#))	Coating	Gloss after aging (GU)			Aging gloss loss rate (%)	60° gloss before aging (GU)
		20°	60°	85°		
0	Without microcapsules	3.80	23.80	39.30	-	24.20
4	Basswood coating with SA-MCs	1.40	11.70	25.10	50.84	13.90
5		1.10	8.10	19.30	65.97	10.60
9	Basswood coating with GA-MCs	0.60	3.80	9.50	84.03	5.10
10		0.60	3.50	5.40	85.29	4.70
